# The changed endemic pattern of human adenovirus from species C to B among children in 2022–2024 in Shenzhen, China

**DOI:** 10.1038/s41598-026-36811-9

**Published:** 2026-01-21

**Authors:** Dan-dan Niu, Zhen Zhang, Zhi-gao Chen, Qiu-ying Lv, Ting-ting Liu, Ni-xuan Chen, Ying-ying Li, Ying Sun, Chao Li, Shun-wu Huang, Yan-peng Cheng, Hong-lin Wang, Ying Wen, Xin-dong Zhang, Xuan Zou, Jian-hua Lu, Yi-xiong Chen, Xiao-lu Shi, Shi-song Fang, Tie-jian Feng

**Affiliations:** 1 Shenzhen Center for Disease Control and Prevention, No. 8 Longyuan Road, Shenzhen, 518055 Guangdong China; 2 Baoan Center for Disease Control and Prevention, No. 3 Haixiu Road, Shenzhen, 518100 Guangdong China; 3 Shenzhen Research Center for Communicable Disease Control and Prevention, Chinese Academy of Medical Sciences, Shenzhen, 518055 Guangdong China

**Keywords:** Human adenovirus, Molecular epidemiology, Children, Shenzhen, Diseases, Health care, Medical research, Microbiology

## Abstract

Respiratory human adenovirus (HAdV) infections pose a significant threat to children’s health. This study investigated the prevalence and molecular epidemiology of respiratory HAdV in children from both hospital and community settings in Shenzhen between 2022 and 2024. Children under 14 years of age with suspected acute respiratory infections were enrolled from eight hospitals between November 2022 and June 2024, as well as from communities between October 2023 and April 2024 in Shenzhen. Respiratory specimens were collected and tested for HAdV. The positive rates of HAdV across different groups were compared using Pearson’s chi-square test with the Bonferroni correction, and phylogenetic analysis was performed on the detected HAdV strains. The positivity proportion of HAdV among children in hospital was 16.7% (83/498) between September 2023 and June 2024, predominantly involving HAdV-B3 (95.2%, 20/21). This rate was significantly higher than that observed from October 2022 to June 2023 (3.4%, 11/321) (χ² = 33.676, *P* < 0.001), during which HAdV-C1 was the predominant subtype (77.8%, 7/9). The positive proportion of HAdV in hospitalized children was higher than that in outpatient children (χ2 = 0.275, *P* < 0.001), and the positive proportion of HAdV in boys was lower than that in girls in community (χ2 = 4.843, *P* = 0.028). No significant difference in the mean age was observed between children infected with HAdV-B and those with HAdV-C (t = 0.670, *P* = 0.509). The Shenzhen-2023-2-ILI-P600 strain exhibited a deletion in the 362–365 aa region of its Penton base gene. Analysis of global HAdV-C1 strains revealed that aa differences at positions 339 and 472 of the Fiber gene were closely linked to specific evolutionary branches. Global HAdV-B3 strains, while highly conserved, showed branch-associated aa differences at positions 168, 403, and 541 of the Hexon gene. The rise in HAdV positivity among pediatric patients in Shenzhen hospitals from late 2022 to late 2023 coincided with a shift in the dominant type from HAdV-C1 to HAdV-B3. Branch-specific aa differences were identified in the Fiber gene of HAdV-C1 and the Hexon gene of HAdV-B3.

**Supplementary Information:**

The online version contains supplementary material available at 10.1038/s41598-026-36811-9.

## Introduction

Human adenovirus (HAdV) is a common pediatric respiratory pathogen responsible for a spectrum of illnesses, from the common cold and bronchitis to pneumonia. It can also lead to severe complications, posing a significant threat to child health. Epidemiological data indicated that HAdV accounted for approximately 3–7% of all acute respiratory infections (ARIs) in children^1^. HAdV was medium-sized (70–100 nm), nonenveloped viruses characterized by an icosahedral nucleocapsid that enclosed a double-stranded linear DNA genome of approximately 34–36 kbp. Based on genomic characteristics, HAdV are classified into seven species (A-G). Among these, species B (e.g., types 3, 7, 55), C (e.g., types 1, 2, 5), and E (type 4) are most frequently associated with ARIs in children^2,3^.

The COVID-19 pandemic in 2020 profoundly impacted global health. Research indicates that the implementation of non-pharmaceutical interventions (NPIs) significantly altered the circulation dynamics of multiple respiratory pathogens, including HAdV, influenza virus, and *Mycoplasma pneumoniae*^4,5^. Following the COVID-19 outbreak, the prevalence of HAdV generally exhibited an initial decrease followed by a subsequent increase^6,7^. According to surveillance data from Beijing, the previously dominant strains HAdV-B3 and HAdV-B7 decreased sharply in 2021, with HAdV-B7 even going undetected. In contrast, HAdV-C1 emerged as the new dominant strain. Furthermore, the proportion of unclassified strains also increased^8^. These results suggest that the implementation of NPIs may have been a key driver of shifts in dominant HAdV strains, which may have contributed to a severe outbreak of respiratory HAdV infection among children aged 3–6 years in Hokkaido, Japan, in 2023^9^. Surveillance data from South Korea also indicated an increase in HAdV positivity among children aged 0–6 and 7–12 years in 2023. Molecular epidemiological analysis revealed that the dominant serotypes shifted from HAdV-C1 and C2 during the COVID-19 pandemic to HAdV-B3 in the post-pandemic period^10^. A multicenter retrospective study in Italy also identified HAdV-B3 as the predominant serotype during 2022–2023^11^. A similar shift in the HAdV epidemic profile was observed in China. Surveillance data showed a rapid increase in the positivity proportion of HAdV species B beginning in October 2023. This surge was temporally associated with cluster outbreaks in schools, suggesting that transmission among school-aged children may have been a key driver of the high HAdV incidence in China^12^.

Shenzhen, a megacity in southern China characterized by a dense population and high mobility, faces significant risks of infectious disease importation and spread^13^. It remained unknown how the COVID-19 pandemic influenced the prevalence, molecular epidemiology, and clinical profile of respiratory HAdV in children in Shenzhen. Therefore, this study systematically analyzed the epidemiological and etiological characteristics of pediatric HAdV infection across hospital and community settings between 2022 and 2024, to generate evidence for refining targeted control measures.

## Methods

### Ethical approval statement

The protocol for this surveillance project was reviewed and approved by the Ethics Committee of the Shenzhen Center for Disease Control and Prevention (Approval No. SZCDCLL-[2023]027 A). Informed consent was obtained from all participating children or their guardians prior to the collection of respiratory samples and demographic data. All experimental procedures were performed in accordance with the relevant guidelines and regulations.

### Participants

Children under 14 years of age presenting with suspected ARIs were consecutively enrolled from eight hospitals across five districts in Shenzhen between November 2022 and April 2024 (data from July and August 2023 were excluded). The participating departments predominantly comprised the emergency department, fever clinic, respiratory department, pediatrics department, and intensive care unit (ICU). The case definition of ARI was based on the 2016 clinical practice guidelines issued by the Chinese Thoracic Society^14^. Participants were excluded if they had non-infectious diseases (e.g., asthma or cancer), lacked signed informed consent, or had missing important data.

This study also enrolled children under 14 years of age from a community cohort in four districts of Shenzhen between October 2023 and April 2024, selected through a three-stage stratified cluster random sampling method. In the first stage, three communities—representing an urban village, an old community, and an urbanized community—were randomly selected at a 1:1:1 ratio. In the second stage, buildings within each chosen community were randomly identified for investigation. In the third stage, all children from households residing in the selected buildings were included by cluster random sampling. Trained investigators administered a structured questionnaire through face-to-face interviews at least once per month. The baseline survey collected information on age, gender, ethnicity, height, weight, number of family members, and home address. Follow-up surveys recorded the follow-up date, occurrence of any symptoms within the preceding two weeks, and the number of follow-up visits completed. If participants or their guardians reported suspected respiratory symptoms, the case was further verified through consultation with a professional physician. After each survey, quality control was conducted by dedicated staff to check for missing questionnaire content. Children who declined to cooperate with investigators or whose questionnaire data contained logical inconsistencies were excluded from the study.

### Sample collection and pathogen detection

Respiratory samples, including nasopharyngeal swabs, sputum, and bronchial or alveolar lavage fluid, were collected from pediatric patients within 72 h of presentation upon enrollment and prior to any treatment^15^. A stratified sampling approach was applied in hospitals: nasopharyngeal swabs were prioritized for upper respiratory tract infections, while bronchial or alveolar lavage fluid was preferred for lower respiratory tract infections, including pneumonia. To ensure balanced distribution and representativeness, each hospital collected at least five samples weekly, with at least one sample obtained on each of Tuesday, Thursday, and Saturday. When sample availability was limited, all eligible specimens were included. In the community cohort, nasopharyngeal swabs were collected during each scheduled questionnaire survey.

The collected samples were stored in transport tubes and delivered to the Shenzhen Center for Disease Control and Prevention under cold-chain conditions for pathogen detection. A sample submission form was completed for each specimen, documenting the collection time, sample type, and other relevant information. Nucleic acids were extracted using a viral nucleic acid extraction kit on an automated extractor. Pathogen detection was performed with a multiplex PCR-based rapid detection kit capable of identifying 22 respiratory pathogens, including: influenza virus (H1N1 and H3N2 subtypes of influenza A; Victoria and Yamagata lineages of influenza B), SARS-CoV-2, rhinovirus, human adenovirus (HAdV), respiratory syncytial virus, parainfluenza virus (types 1–4), human metapneumovirus, bocavirus, coronaviruses (OC43, 229E, HKU1, and NL63), *Mycoplasma pneumoniae*, *Streptococcus pneumoniae*, enterovirus, and *Chlamydia pneumoniae*. All laboratory procedures were conducted in compliance with the Regulations on Biosafety Management and the Standard Operating Procedures of the National Pathogenic Microorganism Laboratories.

### Determination of the sequences of Penton base, Hexon, and Fiber genes of HAdV

Three target gene regions—Penton base (1253 bp), Hexon (1685 bp), and Fiber (1153 bp)—were amplified from HAdV-positive samples using published universal primers and standard protocols^16^. This detection technique covers 22 common HAdV serotypes (A18, B3, B7, B11, B14, B16, B21, B34, B35, B50, B55, B66, B68, C1, C2, C5, C6, D19, D37, E4, F41, G52). The PCR products from HAdV-positive samples were subjected to bidirectional sequencing of the three target genes by Sangon Biotech (Shanghai) Co., Ltd. to ensure sequence accuracy.

### Phylogenetic analysis

Sequencing data were processed using Sequencher 5.0 software (Genecode, USA) to assemble and edit the Loop2 region (960 bp) of the Hexon gene, along with the complete nucleotide sequences of the Penton base, Hexon, and Fiber genes. The curated Loop2 region and the three full gene sequences were then extracted and saved. The Basic Local Alignment Search Tool (BLAST) from the National Center for Biotechnology Information (NCBI) GenBank database was employed for homologous sequence alignment. Reference sequences of the three target genes from global HAdV strains were downloaded from NCBI and combined with the sequences obtained in this study to construct phylogenetic trees. Multiple sequence alignment was performed with MAFFT 7.311 (http://mafft.cbrc.jp/alignment/software/), and the results were visualized using DNAMAN 19.0. Phylogenetic and genetic distance analyses were conducted in MEGA 11.0. Trees were inferred using both the neighbor-joining (NJ) method under the Kimura 2-parameter nucleotide substitution model and the maximum likelihood (ML) method under the Tamura–Nei model. Branch support was assessed with 1000 bootstrap replicates^17^. Sequence variations in the nucleotide (nt) and amino acid (aa) sequences of the three HAdV genes were analyzed using Bioedit software (version 7.0.4.1).

### Statistical analysis

Questionnaire data were double-entered into a standardized database using EpiData 4.6 and Microsoft Excel 2010, with any missing information supplemented by contacting the relevant physicians or investigators. Descriptive analyses were performed by expressing qualitative variables as frequency and percentage, and quantitative variables as mean ± standard deviation (for normally distributed data) or median and interquartile range (M (Q1, Q3), for skewed data). Children were categorized into two groups for analysis: those under 5 years old and school-aged children (5–14 years old). Group differences in mean age were compared using an independent samples t-test, while differences in pathogen positivity rates were assessed with Pearson’s chi-square test and the Bonferroni correction. All statistical analyses were conducted in R 4.1.2, with a two-sided p-value < 0.05 considered statistically significant.

## Results

### Demographic characteristics of participants

From October 2022 to June 2023, a total of 321 children with ARIs were enrolled from hospitals. The median age was 3.8 years (IQR: 1.0–6.6), with 61.1% under 5 years of age. Males accounted for 63.2% of the participants, and 39.6% were hospitalized cases. From September 2023 to June 2024, a total of 498 children with ARIs were enrolled. The participant had a median age of 5.0 years (IQR: 2.8–7.0), with 46.8% under 5 years of age, 57.6% male, and 44.4% hospitalized.

From September 2023 to April 2024, a total of 1,533 children were enrolled from community settings. The median age was 9.0 years (IQR: 6.0–11.0). Among them, 9.1% were under 5 years of age, 50.7% were male, and 6.3% presented with symptoms (Table 1).

**Table 1:** Sociodemographic and clinical characteristics of the participants.

Groups		Children in hospital (2022.10-2023.6)			Children in hospital (2023.9-2024.6)			Children in community (2023.9-2024.4)			
		Total	Outpatient	Inpatient	Total	Outpatient	Inpatient	Total	With respiratory symptom	Without respiratory symptom	Missing
Total		321	194 (60.4)	127 (39.6)	498	277 (55.62)	221 (44.38)	1533	97 (6.3)	1366 (89.1)	90 (5.9)
Median age (years)		3.8 (1.0, 6.6)	5.0 (3.0, 7.0)	0.9 (0.1, 3.8)	5.0 (2.8, 7.0)	5.3 (3.2, 7.0)	4.8 (2.3, 7.0)	9.0 (6.0, 11.0)	8.0 (6.0, 11.0)	9.0 (6.0, 11.0)	10.0 (8.0, 11.0)
Age (years)	< 5	196 (61.1)	89 (45.4)	107 (54.6)	233 (46.8)	120 (51.5)	113 (48.5)	140 (9.1)	129 (92.1)	11 (7.9)	0 (0.0)
	5–14	125 (38.9)	105 (84.0)	20 (16.0)	265 (53.2)	157 (59.3)	108 (40.8)	1393 (90.9)	1237 (88.8)	86 (6.2)	70 (5.0)
Sex	Male	203 (63.2)	123 (60.6)	80 (39.4)	287 (57.6)	156 (54.4)	131 (45.6)	777 (50.7)	46 (5.9)	681 (87.6)	50 (6.4)
	Female	118 (36.8)	71 (60.2)	47 (39.8)	211 (42.4)	121 (57.4)	90 (42.7)	756 (49.3)	51 (6.7)	685 (90.6)	20 (2.6)
Months of sampling	Sept.	0 (0.0)	0 (0.0)	0 (0.0)	2 (0.4)	1 (50.0)	1 (50.0)	1 (0.0)	0 (0.0)	1 (100.0)	0 (0.0)
	Oct.	2 (0.6)	0 (0.0)	2 (100.0)	44 (8.8)	27 (61.4)	17 (38.6)	220 (14.4)	15 (6.8)	194 (88.2)	11 (5.0)
	Nov.	16 (5.0)	7 (43.8)	9 (56.3)	59 (11.9)	22 (37.3)	37 (62.7)	219 (14.3)	20 (9.1)	188 (85.8)	11 (5.0)
	Dec.	17 (5.3)	1 (5.9)	16 (94.1)	62 (12.5)	1 (1.6)	61 (98.4)	220 (14.4)	16 (7.3)	195 (88.6)	9 (4.1)
	Jan.	1 (0.3)	1 (100.0)	0 (0.0)	69 (13.9)	48 (69.6)	21 (30.4)	221 (14.4)	20 (9.0)	194 (87.8)	7 (3.2)
	Feb.	40 (12.5)	33 (82.5)	7 (17.5)	61 (12.3)	39 (63.9)	22 (36.1)	215 (14.0)	5 (2.3)	201 (93.5)	9 (4.2)
	Mar.	65 (20.2)	45 (69.2)	20 (30.8)	66 (13.3)	42 (63.6)	24 (36.4)	221 (14.4)	15 (6.8)	194 (87.8)	12 (5.4)
	Apr.	71 (22.1)	41 (57.7)	30 (42.3)	66 (13.3)	48 (72.7)	18 (27.3)	216 (14.1)	6 (2.8)	199 (92.1)	11 (5.1)
	May	69 (21.5)	36 (52.2)	33 (47.8)	65 (13.1)	45 (69.2)	20 (30.8)	-	-	-	-
	Jun.	40 (12.5)	30 (75.0)	10 (25.0)	4 (0.8)	4 (100.0)	0 (0.0)	-	-	-	-

The data were presented as the number (%) unless otherwise indicated. The total HAdV positive proportion in each column was calculated using the total number of cases in each column as the denominator, and the HAdV positive proportion in each column was calculated using the number of cases in each row as the denominator.

### The positive proportions of HAdV among children

From October 2022 to June 2023, the positive proportion of HAdV among children in hospitals in Shenzhen was 3.4% (11/321). Of these HAdV-positive cases, six cases were co-infected with other pathogens: three with influenza A virus (H1N1), two with *Streptococcus pneumoniae*, one with *Bordetella pertussis*, and one with human parainfluenza virus type 3.

Between September 2023 and June 2024, the overall positive proportion of HAdV among pediatric hospital patients was 16.7% (83/498), which was significantly higher than the proportion observed between October 2022 and June 2023 (χ² = 33.676, *P* < 0.001). Significant differences in the HAdV-positive proportion were also observed across different months (χ² = 100.309, *P* < 0.001), and it was higher among inpatients than outpatients (χ² = 0.275, *P* < 0.001), as well as in children presenting with a sore throat compared to those without (χ² = 14.224, *P* < 0.001). Additionally, 17 cases were identified as co-infections with other pathogens, specifically 16 with *Streptococcus pneumoniae*, and one each with human rhinovirus, enterovirus, and human metapneumovirus.

**Fig. 1:**
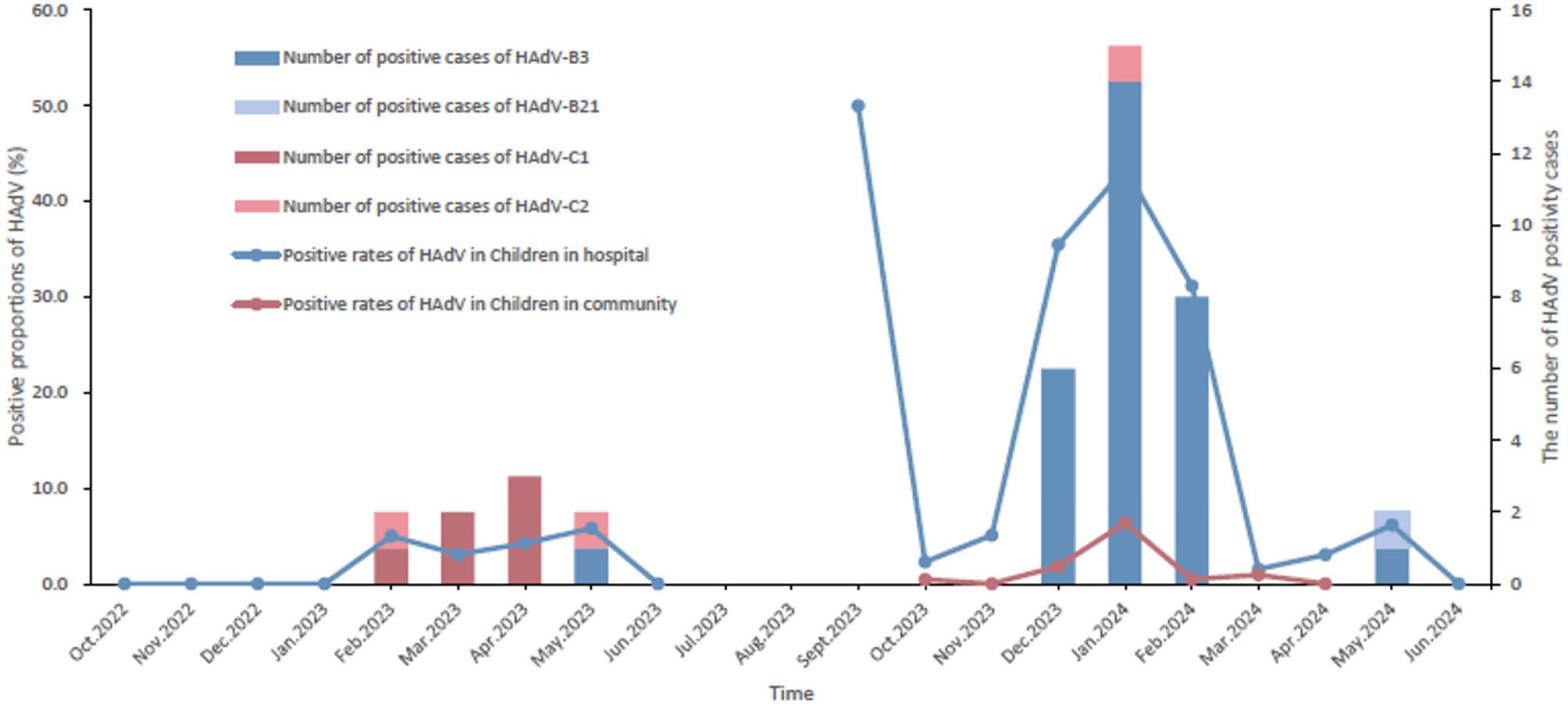
Temporal trends of HAdV positive proportions and circulating types among children from hospital and community in Shenzhen in 2022–2024.

From September 2023 to April 2024, the positive proportion of HAdV among children in the community was 1.5% (22/1517), which was significantly lower than that in the hospital setting during a comparable period (χ² = 8.209, *P* = 0.004). The positivity proportion varied significantly by month (χ² = 7.566, *P* = 0.006), peaking at 6.4% in January 2024. It was also significantly higher in girls than in boys (χ² = 4.843, *P* = 0.028). Regarding clinical symptoms, the HAdV positivity proportion was markedly higher in symptomatic children. Specifically, it was elevated in children with any respiratory symptoms (5.3%, 5/95) compared to asymptomatic individuals (1.2%, *P* = 0.010), and in those presenting with fever (*P* = 0.001), cough and expectoration (*P* = 0.015), or nasal discharge (*P* = 0.015). Furthermore, six co-infection cases were identified, involving one case each of *Mycoplasma pneumoniae*, rhinovirus, influenza A virus (H3 subtype), human metapneumovirus, *Bordetella pertussis*, and human respiratory syncytial virus (group B) (Table 2; Fig. 1).

**Table 2:** Comparison of HAdV positive proportions in different groups among children in hospitals and community settings in Shenzhen from 2022–2024.

Groups		HAdV infections of children in hospital (2022.10-2023.6)					HAdV infections of children in hospital (2023.9-2024.6)					HAdV infections of children in community (2023.9-2024.4)				
		Total	Positive numbers	Positive proportions (%)	χ^2^	*P*	Total	Positive numbers	Positive proportions (%)	χ^2^	*P*	Total	Positive numbers	Positive proportions (%)	χ^2^	*P*
Total		321	11	3.4			498	83	16.7			1517	22	1.5		
Age (Years)	< 5	196	5	2.6	-	0.349	233	34	14.6	1.357	0.244	138	2	1.4	-	1.000
	5–14	125	6	4.8			265	49	18.5			1379	20	1.5		
Sex	Male	203	7	3.4	0.001	0.978	287	45	15.7	0.475	0.491	767	6	0.8	4.843	0.028
	Female	118	4	3.4			211	38	18.0			750	16	2.1		
Case type	Outpatient	194	9	4.6	2.417	0.120	277	44	15.9	0.275	< 0.001	-	-	-	-	-
	Inpatient	127	2	1.6			221	39	17.6			-	-	-	-	-
Respiratory symptoms	Yes	-	-	-	-	-	-	-	-	-	-	95	5	5.3	-	0.010
	No	-	-	-	-	-	-	-	-	-	-	1352	16	1.2		
Fever	Yes	243	8	3.3	0.053	0.817	345	63	18.3	1.673	0.196	31	4	12.9	-	0.001
	No	78	3	3.8			61	7	11.5			1416	17	1.2		
Cough and sputum	Yes	56	2	3.6	0.004	0.948	123	14	11.4	3.597	0.058	68	4	5.9	-	0.015
	No	265	9	3.4			295	56	19.0			1379	17	1.2		
Nasal obstruction	Yes	84	4	4.8	0.573	0.449	62	7	11.3	1.554	0.212	30	1	3.3	-	0.358
	No	237	7	3.0			356	63	17.7			1417	20	1.4		
Running nose	Yes	57	3	5.3	0.632	0.427	89	14	15.7	0.084	0.772	37	3	8.1	-	0.015
	No	264	8	3.0			329	56	17.0			1410	18	1.3		
Sore throat	Yes	4	0	0.0	0.281	0.596	231	53	22.9	14.224	< 0.001	24	1	4.2	-	0.298
	No	317	11	3.5			187	17	9.1			1423	20	1.4		
Diarrhea	Yes	9	0	0.0	0.281	0.596	0	0	0.0	-	-	1	0	0.0	-	1.000
	No	312	11	3.5			418	70	16.7			1446	21	1.5		
Months of sampling	Sept.	-	-	-	-	-	2	1	50.0	100.309	< 0.001	-	-	-	-	-
	Oct.	2	0	0.0	1.644	0.200	44	1	2.3			218	1	0.5	7.566	0.006
	Nov.	16	0	0.0			59	3	5.1			218	0	0.0		
	Dec.	17	0	0.0			62	22	35.5			218	4	1.8		
	Jan.	1	0	0.0			69	30	43.5			220	14	6.4		
	Feb.	40	2	5.0			61	19	31.1			214	1	0.5		
	Mar.	65	2	3.1			66	1	1.5			215	2	0.9		
	Apr.	71	3	4.2			66	2	3.0			214	0	0.0		
	May	69	4	5.8			65	4	6.2			-	-	-		
	Jun.	40	0	0.0			4	0	0.0			-	-	-		

Preliminary type identification and Hexon Loop 2 region aa identity analysis of HAdV.

**Fig. 2:**
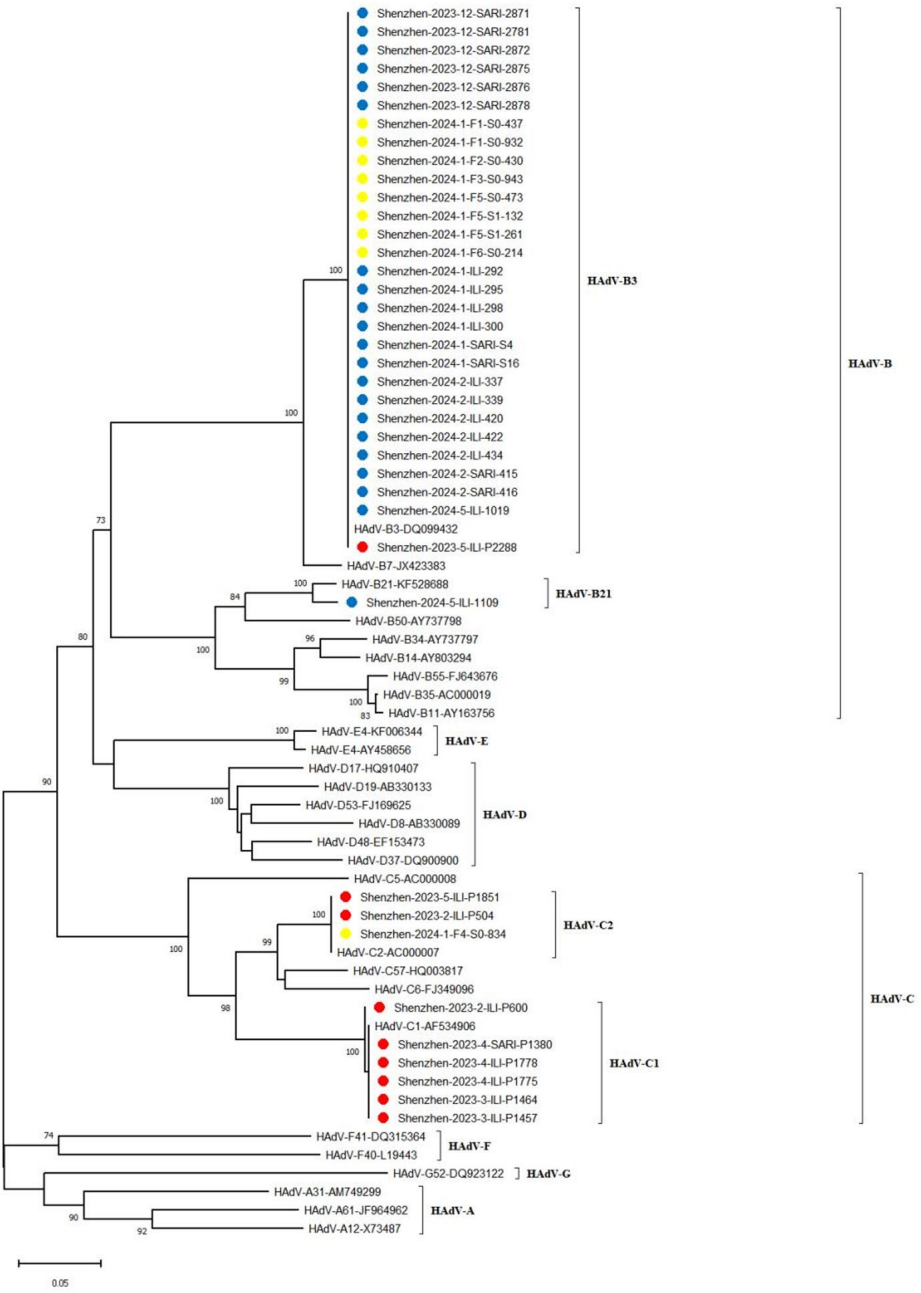
Phylogenetic tree of Hexon Loop 2 gene of HAdV positive samples from children in Shenzhen hospital and community in 2022–2024. Red circle: Sequences of HAdV positive strains in hospital children from October 2022 to June 2023; blue circle: Sequences of HAdV positive strains in hospital children from September 2023 to June 2024; yellow circle: sequences of HAdV positive strains in community children from September 2023 to April 2024.

The Loop 2 region was successfully amplified in 40 HAdV-positive strains, comprising 9 from children in hospital (October 2022–June 2023), 22 from children in hospital (September 2023–June 2024), and 9 from community children (September 2023–April 2024). BLAST analysis of the Loop 2 sequences identified four serotypes belonging to species B and C: HAdV-B3, -B21, -C1, and -C2. A notable shift in the predominant serotype was observed in the hospital setting. In the earlier period (2022–2023), HAdV-C1 was dominant (66.7%, 6/9), with HAdV-C2 (*n* = 2) and HAdV-B3 (*n* = 1) also detected. In the subsequent period (2023–2024), HAdV-B3 became the predominant type, accounting for 95.5% (21/22) of cases, with one case of HAdV-B21. Similarly, HAdV-B3 was the dominant serotype (88.9%, 8/9) among community children, with one HAdV-C2 case identified (Figs. 1 and 2, and Additional file: Supplement Table 1).

The mean age of children infected with HAdV-C (Oct 2022–Jun 2023) and HAdV-B (Sep 2023–Jun 2024) in the hospital setting was 5.2 ± 2.1 years and 5.8 ± 2.2 years, respectively, with no statistically significant difference between the two groups (t = 0.670, *P* = 0.509). Similarly, the hospitalization rates showed no significant difference, at 12.5% (1/8) for the HAdV-C group and 45.5% (10/22) for the HAdV-B group (*P* = 0.199).

Sequence analysis of the Loop 2 region revealed high intra-serotype conservation. Both HAdV-C2 and HAdV-B3 exhibited 100% nt and aa identity among their respective strains. Similarly, HAdV-C1 strains showed near-complete identity (nt: 99.94%, aa: 100%). In contrast, inter-serotype comparisons demonstrated substantial genetic divergence. The identities between HAdV-C1 and HAdV-C2 were 87.4% (nt) and 81.8% (aa); between HAdV-C1 and HAdV-B3, they were 70.8% (nt) and 58.8% (aa); and between HAdV-C2 and HAdV-B3, 72.5% (nt) and 62.6% (aa).

### Phylogenetic and genetic variation analysis of the major capsid genes in global HAdV-C1 strains

The gene-specific databases for Penton base, Hexon, and Fiber each comprised 78 global HAdV-C1 sequences. In addition to the six strains identified in this study from children in hospital (October 2022 to June 2023), the databases included two sequences from Qinghai Province and Shanghai, China (2016–2018), and 70 sequences from other countries, including the United States, Canada, Egypt, Argentina, and Singapore (2012–2020) (Additional file: Supplement Table 2).

**Fig. 3:**
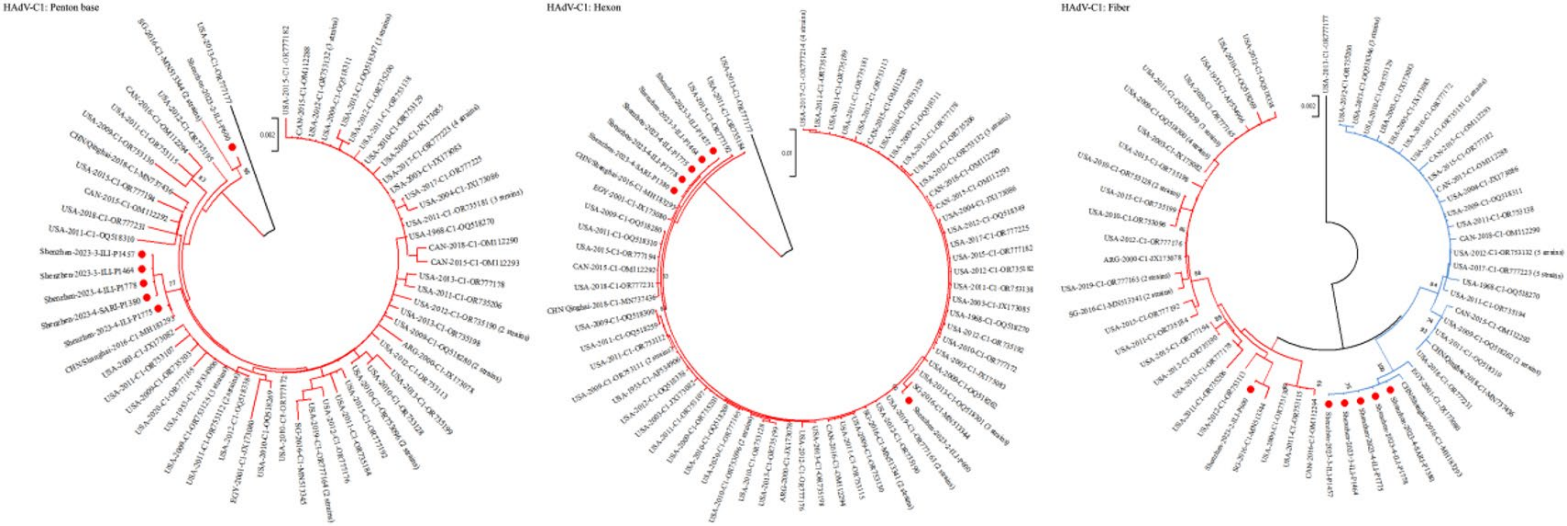
Phylogenetic tree of Penton base, Hexon and Fiber genes of global HAdV-C1 strains. Red circle: Sequences of HAdV positive strains among children in hospital from October 2022 to June 2023. Numbers in parentheses denoted the count of strains from the same country and year with identical sequences. Clades 1 and 2 were colored in red and blue, respectively.

Phylogenetic trees were constructed from the three gene databases using both NJ and ML methods. As the two methods produced topologically congruent trees, only the NJ tree was presented. Phylogenetic analysis revealed that, with the exception of one strain isolated in the United States in 2013 (GenBank: OR777177), globally circulating HAdV-C1 strains formed a predominant lineage (Clade 1) in both the Penton base and Hexon genes, which was prevalent from 1953 to 2020. This clade included strains from Shanghai and Qinghai Province in China, as well as from the United States, Canada, Singapore, Egypt, and Argentina. In contrast, the Fiber gene phylogeny suggested the existence of two distinct evolutionary branches (Clade 1 and Clade 2, Fig. 3). Among them, Clade 1 was first identified in the United States in 1953 and was subsequently detected in the same country (2003–2020) as well as in Argentina (2000), Singapore, and Canada (2016). Clade 2, prevalent between 1968 and 2019, has been identified in Shanghai and Qinghai Province of China, alongside the United States, Canada, Egypt, and Singapore.

Phylogenetic analysis placed all six HAdV-C1 strains from Shenzhen (2022–2023) within Clade 1 of the Penton base and Hexon genes. These strains exhibited high sequence homology (> 99.2% nt, > 99.3% aa) with earlier Chinese strains (2016–2018) and similarly high identity (> 99.0% nt/aa) with other global Clade 1 strains (1953–2020). In the Fiber gene, however, a divergence was observed: one strain (Shenzhen-2023-2-ILI-P600) clustered in Clade 1, showing > 99.3% nt and > 98.6% aa homology with other Clade 1 strains worldwide. The remaining five strains fell into Clade 2, where they displayed > 99.3% nt and > 99.7% aa homology with Chinese strains and > 99.3% nt/aa homology with other global Clade 2 strains (1968–2019) (Additional file: Supplement Table 3). Based on Penton base and Hexon gene, the average p-distance within Clade 1 was 0.003 and 0.002, respectively. Based on Fiber gene, the average p-distance within Clade 1 and Clade 2 was 0.004 and 0.003, respectively, and the average p-distance between Clade 1 and Clade 2 was 0.008 (Additional file: Supplement Table 4).

Comparative amino acid sequence analysis identified deletions in the Penton base gene, specifically a 362–365 aa deletion in the Shenzhen-2023-2-ILI-P600 strain and a 358–359 aa deletion in the CHN/Qinghai strain. In contrast, the Hexon and Fiber genes were highly conserved across all global HAdV-C1 strains, with no insertions or deletions detected. Although most amino acid mutations were scattered, specific variations at positions 339 and 472 in the Fiber gene were strongly associated with distinct evolutionary branches (Additional file: Supplement Table 5).

As the two major neutralization antigens of HAdV, Hexon and Fiber were further examined for sequence variations. The Hexon protein was completely conserved in all studied strains, showing no amino acid changes (Additional file: Supplement Fig. 2). Conversely, the knob domain of the Fiber protein exhibited six amino acid substitutions relative to the reference strain AF534906 (Additional file: Supplement Fig. 2).

### Phylogenetic and genetic variation analysis of the major capsid genes in global HAdV-B3 strains

Further phylogenetic analysis was performed by constructing three gene sequence databases for Penton base (*n* = 108), Hexon (*n* = 156), and Fiber (*n* = 148) to compare the HAdV-B3 strains from this study with global strains. The databases included 30 strains from our study (covering both hospital and community cases from 2022 to 2024), supplemented by 75 sequences from eight provinces in China (1964–2019) and 55 sequences from four other countries (1988–2020) (Additional file: Supplement Table 6).

**Fig. 4:**
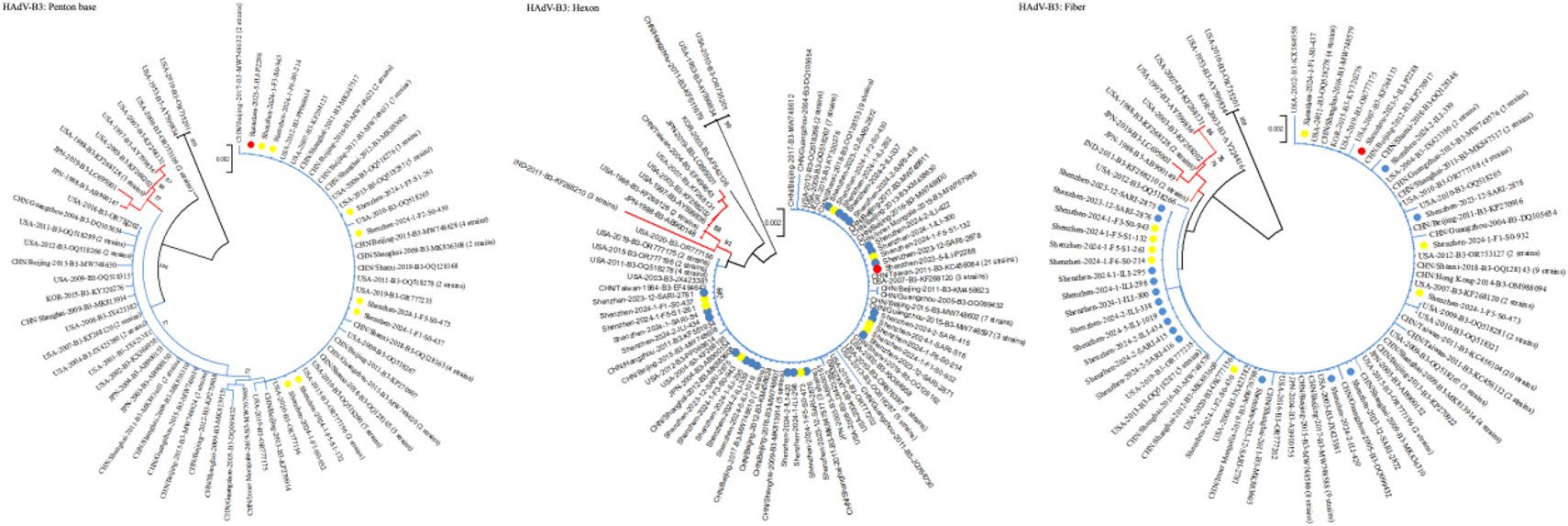
Phylogenetic trees of Penton base, Hexon and Fiber genes of global HAdV-B3 strains. Red circle: sequences of HAdV positive strains in hospital children from October 2022 to June 2023; blue circle: sequences of HAdV positive strains among children in hospital from September 2023 to June 2024; yellow circle: sequences of HAdV positive strains among children in community from September 2023 to April 2024. Numbers in parentheses denoted the count of strains from the same country and year with identical sequences. Clades 1 and 2 were colored in red and blue, respectively.

Phylogenetic analysis was performed on the three gene databases using both NJ and ML methods. As the resulting trees yielded identical topologies, only the NJ tree is displayed. The phylogenetic reconstruction based on the three genes delineated global HAdV-B3 strains into two major evolutionary clades (Clade 1 and Clade 2, Fig. 4). Phylogenetic analysis of the Penton base, Hexon, and Fiber genes consistently delineated two primary clades with distinct spatiotemporal distributions. Clade 1, predominantly circulating from 1988 to 2019, was primarily identified in the United States and Japan across all three genes. In contrast, Clade 2, first detected in the United States in 1988, exhibited a broader geographic range. It subsequently spread to multiple countries including Japan, South Korea, and India, and was widely reported across diverse regions of China, such as Beijing, Shanghai, Guangdong, and Shanxi, from the early 2000s until 2020.

All HAdV-B3 strains from this study consistently clustered within Clade 2 across the Penton base, Hexon, and Fiber genes. They exhibited high sequence homology with other Clade 2 strains: in the Penton base gene, > 99.8% nt and > 99.5% aa with Chinese strains (2004–2019), and > 99.9% nt and > 99.8% aa with international strains (1988–2020). In the Hexon gene, homology was > 98.6% nt and > 97.4% aa with all Clade 2 strains. For the Fiber gene, homology reached > 99.8% nt and > 99.4% aa with Chinese strains and > 98.3% nt and > 96.5% aa with strains from other countries (Additional file: Supplement Table 3). Genetic distance analysis based on the Penton base, Hexon, and Fiber genes showed that the average nucleotide p-distance within Clade 1 was 0.002, 0.003, and 0.002, respectively, and within Clade 2 was 0.001, 0.0002, and 0.0008, respectively. The inter-clade distance between Clade 1 and Clade 2 was 0.005, 0.004, and 0.003, respectively (Additional file: Supplement Table 4).

Comparative amino acid sequence analysis of the three genes revealed high conservation among globally circulating HAdV-B3 strains, with no insertions or deletions identified. While most aa variations were scattered, specific differences at positions 168, 403, and 541 in the Hexon gene were strongly associated with distinct evolutionary branches (Additional file: Supplement Table 7).

As the two major neutralization antigens of HAdV, Hexon and Fiber were further examined. In our HAdV-B3 strains, the knob domain of the Fiber protein exhibited eight aa substitutions compared to the reference strain AF534906 (Additional file: Supplement Fig. 1). Additionally, the loop1 and loop2 regions of the Hexon protein showed three and four aa changes, respectively (Additional file: Supplement Fig. 3).

## Discussion

Following the end of the COVID-19 pandemic, the circulation patterns of many respiratory pathogens have changed including HAdV. A significant increase in HAdV positivity was observed in children from hospital in late 2023 compared to late 2022, potentially associated with a shift in the dominant circulating type from HAdV-C1 to HAdV-B3. Higher positivity proportions were found in hospitalized children than in outpatients, and among girls than boys in the community. No significant age difference was observed between children infected with HAdV-B and HAdV-C. Genomic analysis revealed high conservation in three major genes of global HAdV-B3 strains with no insertions or deletions, whereas a deletion was identified in the Penton base gene of HAdV-C1 strains. Amino acid variations at positions 339 and 472 in the Fiber gene of HAdV-C1 and at positions 168, 403, and 541 in the Hexon gene of HAdV-B3 were closely associated with distinct evolutionary branches. These findings provide an important scientific basis for developing targeted HAdV infection control strategies for children in Shenzhen.

The significant increase in HAdV positivity among hospitalized children from late 2022 to late 2023 was potentially linked to a shift in the dominant circulating type from HAdV-C1 to HAdV-B3. This epidemiological pattern is consistent not only with national trends in China^12^, but has also been documented in several other countries^10,11^. Additionally, the rebound in the number of cases attributable to HAdV was also internationally detectable in waste water with evidence supporting the claim that more cases of B3 were seen^18^. Several hypotheses have been proposed to explain the surge in respiratory pathogens, including HAdV, following the COVID-19 pandemic. The leading explanations focus on the immune gap resulting from reduced exposure to pathogens during the pandemic, interruptions in routine vaccination schedules, viral evolution and genetic variation, interactions among co-circulating respiratory pathogens, and the delayed seasonal resurgence of certain infections^19–21^. Our findings suggest that the surge in HAdV cases may be associated with a shift in the dominant circulating strain to B3, though other contributing factors require further investigation.

The positivity proportion of HAdV was higher among hospitalized children than outpatients, and among girls than boys in community settings. By late 2023, HAdV-B3 had become the predominant type in hospitalized children, among whom the positivity proportion was significantly elevated. Phylogenetic analysis revealed clear clustering of these cases, suggesting possible nosocomial transmission^22^. This shift may be explained by several factors. HAdV-B3 is associated with more severe disease, including lower respiratory tract infections^23,24^, consistent with the higher hospitalization rate observed in this study. Additionally, prior exposure to HAdV-C1 in 2022 may have conferred some population immunity, whereas the emerging HAdV-B3 strain could more readily infect susceptible individuals, leading to increased disease severity^25^. Genomic analysis ruled out enhanced virulence due to gene mutations in HAdV-B3 as a contributing factor.

In the community, the higher HAdV-B3 positivity among girls may reflect behavioral or immunological differences. Previous studies indicate that girls often participate earlier in structured group activities (e.g., dance or early education classes), potentially increasing exposure to respiratory viruses^26^.Sex-based differences in immune responses may also play a role, with some evidence suggesting that females are more susceptible to certain viral infections^27^, though underlying mechanisms remain unclear. Future studies combining behavioral surveys with larger community cohorts could help clarify gender-related risk factors.

No significant difference in age distribution was detected between HAdV-B and HAdV-C infections in Shenzhen, contradicting previous reports that HAdV-C typically infects infants and toddlers, while HAdV-B is more common in older children^28^. This discrepancy may reflect recent shifts in circulating strains, blurring previously established age-specific patterns. Potential biases from the limited sample size in this study, as well as confounding factors such as prior immunity or co-infections with other respiratory viruses^29^, may also have influenced age distribution findings and warrant further investigation.

The global HAdV B3 strains were highly conserved. This finding aligned with earlier reports on the genetic stability of HAdV-B3 strains globally, indicating that this type may evolve at a relatively constant rate^30^. Such stability may be attributed to the strong proofreading activity of HAdV DNA polymerase, which significantly enhances the fidelity of viral genome replication^31^. The high degree of sequence conservation in HAdV-B3 provides a promising basis for the development of effective vaccines. The deletion of aa sequences in the Penton base gene of HAdV-C1 were deleted. As the Penton base protein plays a critical role in viral entry into host cells, its structural integrity and receptor-binding capacity are essential for viral infectivity^32^. The 362–365 aa deletion in the Shenzhen strain may induce local conformational changes, potentially altering the binding efficiency to cellular receptors such as fibrin or integrins. In contrast, the 358–359 aa deletion in the Qinghai strain lies within a putative epitope region, which could modulate host immune recognition. These region-specific deletions may reflect localized epidemic pressures acting on HAdV-C1 in China, including host immune selection or antiviral interventions. Future studies employing reverse genetics systems are warranted to elucidate the effects of these deletions on viral replication efficiency, cell tropism, and pathogenicity, supported by structural biology approaches to reveal the underlying molecular mechanisms.

Aa variations at positions 339 and 472 in the Fiber gene of HAdV-C1 and at key sites (168, 403, and 541) in the Hexon gene of HAdV-B3 were closely associated with distinct evolutionary branches, indicating that these changes may contribute to viral adaptation under host immune pressure. As the major capsid protein and carrier of neutralizing epitopes in HAdV^33^, sequence changes in Hexon—particularly at key residues—may directly influence viral immune evasion and cell tropism. Similarly, the Fiber protein, which mediates viral attachment to host cells, may exhibit altered receptor-binding properties due to mutations at positions 339 and 472 in HAdV-C1. The specific aa substitution patterns identified among different clades in this study may serve as valuable genetic markers for molecular surveillance, aiding in tracking transmission routes and detecting emerging variants.

This study has several limitations. First, surveillance data were primarily collected from eight sentinel hospitals in Shenzhen, which may introduce Berkson’s bias. Second, the study period was relatively short and focused on the late phase of the COVID-19 pandemic; thus, it remains unclear whether the replacement of HAdV-C1 by HAdV-B3 represents a sustained trend. Third, the limited sample size may affect the generalizability and reliability of the findings. Nevertheless, we sought to mitigate selection bias through a multicenter retrospective design. Fourth, as community samples were derived from a pediatric cohort, the estimated HAdV positivity rate may be biased—potentially overestimated due to prolonged viral shedding or underestimated if children were in the incubation phase during sampling. Future studies should expand the scope and scale of surveillance and incorporate analyses of neutralizing antibody dynamics and whole-genome sequencing to elucidate the intrinsic drivers underlying shifts in HAdV dominance.

## Conclusions

The rise in HAdV positivity among pediatric patients in Shenzhen hospitals from late 2022 to late 2023 coincided with a shift in the dominant type from HAdV-C1 to HAdV-B3. Globally circulating HAdV-B3 strains showed high genetic conservation, supporting their suitability as targets for vaccine development. Amino acid variations at positions 339 and 472 in the Fiber gene of HAdV-C1 and at residues 168, 403, and 541 in the Hexon gene of HAdV-B3 were associated with distinct phylogenetic clades, reflecting long-term evolutionary dynamics in globally circulating HAdV strains. These findings provide a valuable scientific foundation for public health authorities in Shenzhen to develop evidence-based strategies for the prevention and control of HAdV respiratory infections in children.

## Supplementary Information

Below is the link to the electronic supplementary material.

Supplementary Material 1

## Data Availability

The data that supported the findings of this study were available in the paper and supplementary material of this article.

## Abbreviations

aa: Amino acid
ARIs: Acute respiratory infections
BLAST: Basic Local Alignment Search Tool
HAdV: Human adenovirus
ICU: Intensive care unit
ILI: Influenza-like illness
M(Q1,Q3): Median and interquartile range
ML: Maximum likelihood method
NCBI: National Center for Biotechnology Information
NJ: Neighbor-joining method
NPIs: Non-pharmaceutical interventions
nt: Nucleotide
SARI: Severe acute respiratory infection
