# Supplementary material for: The changed endemic pattern of human adenovirus from species C to B among children in 2022–2024 in Shenzhen, China
Source: Sci Rep. 2026 Jan 21;16:5902. doi: 10.1038/s41598-026-36811-9 (PMC12894712; doi:10.1038/s41598-026-36811-9)
Supplement: Supplementary file 1 — Supplementary Material 1 [file 41598_2026_36811_MOESM1_ESM.docx]

**The changed endemic pattern of human adenovirus from species C to B among children in 2022-2024 in Shenzhen, China**

**Supplement Table 1.** Basic information of human adenovirus positive strains in children in hospitals and community in Shenzhen in 2022-2024

| **Children source** | **Sample number** | **Collection date** | **Children source** | **Respiratory symptom** | **Penton base** | **Hexon** | **Fiber** | **HAdV type** |
| --- | --- | --- | --- | --- | --- | --- | --- | --- |
| Children in hospital (Oct. 2022-Jun. 2023) | Shenzhen-2023-4-SARI-P1380 | 2023-4 | Hospital/SARI | Yes | PV972139 | PV972130 | PV972190 | HAdV-C1 |
|  | Shenzhen-2023-3-ILI-P1457 | 2023-3 | Hospital/ILI | Yes | PV972143 | PV972134 | PV972188 | HAdV-C1 |
|  | Shenzhen-2023-3-ILI-P1464 | 2023-3 | Hospital/ILI | Yes | PV972142 | PV972133 | PV972187 | HAdV-C1 |
|  | Shenzhen-2023-4-ILI-P1775 | 2023-4 | Hospital/ILI | Yes | PV972141 | PV972132 | PV972186 | HAdV-C1 |
|  | Shenzhen-2023-4-ILI-P1778 | 2023-4 | Hospital/ILI | Yes | PV972140 | PV972131 | PV972185 | HAdV-C1 |
|  | Shenzhen-2023-5-ILI-P1851 | 2023-5 | Hospital/ILI | Yes | PV972146 | PV972137 | PV972192 | HAdV-C2 |
|  | Shenzhen-2023-5-ILI-P2288 | 2023-5 | Hospital/ILI | Yes | PV972176 | PV972175 | PV972129 | HAdV-B3 |
|  | Shenzhen-2023-2-ILI-P504 | 2023-2 | Hospital/ILI | Yes | PV972145 | PV972136 | PV972191 | HAdV-C2 |
|  | Shenzhen-2023-2-ILI-P600 | 2023-2 | Hospital/ILI | Yes | PV972144 | PV972135 | PV972189 | HAdV-C1 |
| Children in hospital (Sept. 2023-Jun. 2024) | Shenzhen-2023-12-SARI-2781 | 2023-12 | Hospital/SARI | Yes | × | PV972174 | PV972128 | HAdV-B3 |
|  | Shenzhen-2023-12-SARI-2871 | 2023-12 | Hospital/SARI | Yes | × | PV972173 | × | HAdV-B3 |
|  | Shenzhen-2023-12-SARI-2872 | 2023-12 | Hospital/SARI | Yes | × | PV972172 | PV972127 | HAdV-B3 |
|  | Shenzhen-2023-12-SARI-2875 | 2023-12 | Hospital/SARI | Yes | × | PV972171 | PV972126 | HAdV-B3 |
|  | Shenzhen-2023-12-SARI-2876 | 2023-12 | Hospital/SARI | Yes | × | PV972170 | PV972125 | HAdV-B3 |
|  | Shenzhen-2023-12-SARI-2878 | 2023-12 | Hospital/SARI | Yes | × | PV972169 | PV972124 | HAdV-B3 |
|  | Shenzhen-2024-1-SARI-S4 | 2024-1 | Hospital/SARI | Yes | × | PV972156 | × | HAdV-B3 |
|  | Shenzhen-2024-1-SARI-S16 | 2024-1 | Hospital/SARI | Yes | × | PV972155 | × | HAdV-B3 |
|  | Shenzhen-2024-1-ILI-292 | 2024-1 | Hospital/ILI | Yes | × | PV972160 | × | HAdV-B3 |
|  | Shenzhen-2024-1-ILI-295 | 2024-1 | Hospital/ILI | Yes | × | PV972159 | PV972115 | HAdV-B3 |
|  | Shenzhen-2024-1-ILI-298 | 2024-1 | Hospital/ILI | Yes | × | PV972158 | PV972114 | HAdV-B3 |
|  | Shenzhen-2024-1-ILI-300 | 2024-1 | Hospital/ILI | Yes | × | PV972157 | PV972113 | HAdV-B3 |
|  | Shenzhen-2024-2-ILI-337 | 2024-2 | Hospital/ILI | Yes | × | PV972154 | × | HAdV-B3 |
|  | Shenzhen-2024-2-ILI-338 | 2024-2 | Hospital/ILI | Yes | × | × | PV972112 | HAdV-B3 |
|  | Shenzhen-2024-2-ILI-339 | 2024-2 | Hospital/ILI | Yes | × | PV972153 | PV972111 | HAdV-B3 |
|  | Shenzhen-2024-2-SARI-415 | 2024-2 | Hospital/SARI | Yes | × | PV972150 | PV972108 | HAdV-B3 |
|  | Shenzhen-2024-2-SARI-416 | 2024-2 | Hospital/SARI | Yes | × | PV972149 | PV972107 | HAdV-B3 |
|  | Shenzhen-2024-2-ILI-420 | 2024-2 | Hospital/ILI | Yes | × | PV972152 | PV972110 | HAdV-B3 |
|  | Shenzhen-2024-2-ILI-422 | 2024-2 | Hospital/ILI | Yes | × | PV972151 | × | HAdV-B3 |
|  | Shenzhen-2024-2-ILI-434 | 2024-2 | Hospital/ILI | Yes | × | PV974490 | PV972109 | HAdV-B3 |
|  | Shenzhen-2024-5-ILI-1019 | 2024-5 | Hospital/ILI | Yes | × | PV972148 | PV972106 | HAdV-B3 |
|  | Shenzhen-2024-5-ILI-1109 | 2024-5 | Hospital/ILI | Yes | PX101489 | PX101489 | PX101489 | HAdV-B21 |
| Children in community (Sept. 2023-Apr. 2024) | Shenzhen-2024-1-F1-S0-437 | 2024-1 | Community/Family 1 | No | PV972177 | PV972168 | PV972123 | HAdV-B3 |
|  | Shenzhen-2024-1-F1-S0-932 | 2024-1 | Community/Family 1 | No | PV972178 | PV972167 | PV972122 | HAdV-B3 |
|  | Shenzhen-2024-1-F2-S0-430 | 2024-1 | Community/Family 2 | No | PV972179 | PV972166 | PV972121 | HAdV-B3 |
|  | Shenzhen-2024-1-F3-S0-943 | 2024-1 | Community/Family 3 | No | PV972180 | PV972165 | PV972120 | HAdV-B3 |
|  | Shenzhen-2024-1-F4-S0-834 | 2024-1 | Community/Family 4 | No | PV972147 | PV972138 | PV972193 | HAdV-C2 |
|  | Shenzhen-2024-1-F5-S1-261 | 2024-1 | Community/Family 5 | Yes | PV972181 | PV972162 | PV972117 | HAdV-B3 |
|  | Shenzhen-2024-1-F5-S1-132 | 2024-1 | Community/Family 5 | Yes | PV972182 | PV972163 | PV972118 | HAdV-B3 |
|  | Shenzhen-2024-1-F5-S0-473 | 2024-1 | Community/Family 5 | No | PV972183 | PV972164 | PV972119 | HAdV-B3 |
|  | Shenzhen-2024-1-F6-S0-214 | 2024-1 | Community/Family 6 | No | PV972184 | PV972161 | PV972116 | HAdV-B3 |

**Notes:** SARI: Severe Acute Respiratory Infection; ILI: Influenza-like Illness.

**Supplement Table 2.** The sequences information of global HAdV-C1 strains

| No. | Strain Name in GenBank | Country/Province | Regional abbreviation | Collection Date | Genotype | Accession Number | | | Clade |
| --- | --- | --- | --- | --- | --- | --- | --- | --- | --- |
|  |  |  |  |  |  | Penton base | Hexon | Fiber |  |
| 1 | human/ARG/A15812/2000/1[P1H1F1] | Argentina | ARG | 2000 | HAdV-C1 | JX173078 | JX173078 | JX173078 | Clade 1 |
| 2 | C1ONP03Cu1Jun2015 | Canada Toronto | CAN | 2015 | HAdV-C1 | OM112288 | OM112288 | OM112288 | Clade 2 |
| 3 | C1ONP05Pr1Jan2018 | Canada Toronto | CAN | 2018 | HAdV-C1 | OM112290 | OM112290 | OM112290 | Clade 2 |
| 4 | C1ONP01Pr1Feb2015 | Canada Toronto | CAN | 2015 | HAdV-C1 | OM112292 | OM112292 | OM112292 | Clade 2 |
| 5 | C1ONP02Cu1Apr2015 | Canada Toronto | CAN | 2015 | HAdV-C1 | OM112293 | OM112293 | OM112293 | Clade 2 |
| 6 | C1ONP04Cu1Aug2016 | Canada Toronto | CAN | 2016 | HAdV-C1 | OM112294 | OM112294 | OM112294 | Clade 1 |
| 7 | QH-1665/2018 | China Qinghai | CHN/Qinghai | 2018.11 | HAdV-C1 | MN737436 | MN737436 | MN737436 | Clade 2 |
| 8 | SH2016 | China Shanghai | CHN/Shanghai | 2016.2 | HAdV-C1 | MH183293 | MH183293 | MH183293 | Clade 2 |
| 9 | human/EGY/E13/2001/1[P1H1F1] | Egypt | EGY | 2001.2 | HAdV-C1 | JX173080 | JX173080 | JX173080 | Clade 2 |
| 10 | SG05/HAdvC1/2016 | Singapore | SG | 2016.9 | HAdV-C1 | MN513341 | MN513341 | MN513341 | Clade 1 |
| 11 | SG08/HAdvC1/2016 | Singapore | SG | 2016.1 | HAdV-C1 | MN513344 | MN513344 | MN513344 | Clade 1 |
| 12 | SG09/HAdvC1/2016 | Singapore | SG | 2016.9 | HAdV-C1 | MN513345 | MN513345 | MN513345 | Clade 2 |
| 13 | prototype strain | the United States | USA | 1953 | HAdV-C1 | AF534906 | AF534906 | AF534906 | Clade 1 |
| 14 | human/USA/VT384/2003/1[P1H1F1] | the United States | USA | 2003.2 | HAdV-C1 | JX173082 | JX173082 | JX173082 | Clade 1 |
| 15 | human/USA/VT2672/2003/1[P1H1F1] | the United States | USA | 2003.2 | HAdV-C1 | JX173083 | JX173083 | JX173083 | Clade 2 |
| 16 | human/USA/VT2612/2003/1[P1H1F1] | the United States | USA | 2003.3 | HAdV-C1 | JX173085 | JX173085 | JX173085 | Clade 2 |
| 17 | human/USA/VT13862/2004/1[P1H1F1] | the United States | USA | 2004.1 | HAdV-C1 | JX173086 | JX173086 | JX173086 | Clade 2 |
| 18 | HAdV-C1/USA/8H5/2011 | the United States | USA | 2011.8 | HAdV-C1 | OQ518259 | OQ518259 | OQ518259 | Clade 1 |
| 19 | HAdV-C1/USA/4C3/2009 | the United States | USA | 2009.9 | HAdV-C1 | OQ518262 | OQ518262 | OQ518262 | Clade 2 |
| 20 | HAdV-C1/USA/5T1/2010 | the United States | USA | 2010.2 | HAdV-C1 | OQ518269 | OQ518269 | OQ518269 | Clade 1 |
| 21 | HAdV-C1/USA/9M5/1968 | the United States | USA | 1968.12 | HAdV-C1 | OQ518270 | OQ518270 | OQ518270 | Clade 2 |
| 22 | HAdV-C1/USA/3P2/2009 | the United States | USA | 2009.7 | HAdV-C1 | OQ518280 | OQ518280 | OQ518280 | Clade 2 |
| 23 | HAdV-C1/USA/5M5/2009 | the United States | USA | 2009.12 | HAdV-C1 | OQ518300 | OQ518300 | OQ518300 | Clade 1 |
| 24 | HAdV-C1/USA/11B5/2013 | the United States | USA | 2013.1 | HAdV-C1 | OQ518301 | OQ518301 | OQ518301 | Clade 2 |
| 25 | HAdV-C1/USA/7G4/2011 | the United States | USA | 2011.1 | HAdV-C1 | OQ518310 | OQ518310 | OQ518310 | Clade 2 |
| 26 | HAdV-C1/USA/2C6/2009 | the United States | USA | 2009.4 | HAdV-C1 | OQ518311 | OQ518311 | OQ518311 | Clade 2 |
| 27 | HAdV-C1/USA/9I10/2012 | the United States | USA | 2012.4 | HAdV-C1 | OQ518338 | OQ518338 | OQ518338 | Clade 1 |
| 28 | HAdV-C1/USA/11A9/2013 | the United States | USA | 2013.1 | HAdV-C1 | OQ518346 | OQ518346 | OQ518346 | Clade 2 |
| 29 | HAdV-C1/USA/11A5/2013 | the United States | USA | 2013.1 | HAdV-C1 | OQ518347 | OQ518347 | OQ518347 | Clade 2 |
| 30 | HAdV-C1/USA/9P7/2012 | the United States | USA | 2012.8 | HAdV-C1 | OQ518349 | OQ518349 | OQ518349 | Clade 2 |
| 31 | HAdV-C1/USA/7L7/2011 | the United States | USA | 2011.2 | HAdV-C1 | OR735181 | OR735181 | OR735181 | Clade 2 |
| 32 | HAdV-C1/USA/9E10/2012 | the United States | USA | 2012.4 | HAdV-C1 | OR735182 | OR735182 | OR735182 | Clade 2 |
| 33 | HAdV-C1/USA/8I8/2011 | the United States | USA | 2011.8 | HAdV-C1 | OR735184 | OR735184 | OR735184 | Clade 1 |
| 34 | HAdV-C1/USA/8B5/2011 | the United States | USA | 2011.5 | HAdV-C1 | OR735189 | OR735189 | OR735189 | Clade 2 |
| 35 | HAdV-C1/USA/10B7/2012 | the United States | USA | 2012.1 | HAdV-C1 | OR735190 | OR735190 | OR735190 | Clade 1 |
| 36 | HAdV-C1/USA/9I7/2012 | the United States | USA | 2012.4 | HAdV-C1 | OR735192 | OR735192 | OR735192 | Clade 2 |
| 37 | HAdV-C1/USA/7R7/2011 | the United States | USA | 2011.3 | HAdV-C1 | OR735194 | OR735194 | OR735194 | Clade 2 |
| 38 | HAdV-C1/USA/9P3/2012 | the United States | USA | 2012.7 | HAdV-C1 | OR735195 | OR735195 | OR735195 | Clade 2 |
| 39 | HAdV-C1/USA/11C4/2013 | the United States | USA | 2013.1 | HAdV-C1 | OR735198 | OR735198 | OR735198 | Clade 1 |
| 40 | HAdV-C1/USA/11E2/2013 | the United States | USA | 2013.12 | HAdV-C1 | OR735199 | OR735199 | OR735199 | Clade 1 |
| 41 | HAdV-C1/USA/9K8/2012 | the United States | USA | 2012.5 | HAdV-C1 | OR735200 | OR735200 | OR735200 | Clade 2 |
| 42 | HAdV-C1/USA/5J10/2009 | the United States | USA | 2009.12 | HAdV-C1 | OR735203 | OR735203 | OR735203 | Clade 2 |
| 43 | HAdV-C1/USA/8L7/2011 | the United States | USA | 2011.1 | HAdV-C1 | OR735206 | OR735206 | OR735206 | Clade 1 |
| 44 | HAdV-C1/USA/6B1/2010 | the United States | USA | 2010.2 | HAdV-C1 | OR735211 | OR735211 | OR735211 | Clade 2 |
| 45 | HAdV-C1/USA/6B9/2010 | the United States | USA | 2010.3 | HAdV-C1 | OR753096 | OR753096 | OR753096 | Clade 1 |
| 46 | HAdV-C1/USA/7H9/2011 | the United States | USA | 2011.1 | HAdV-C1 | OR753107 | OR753107 | OR753107 | Clade 2 |
| 47 | HAdV-C1/USA/3A10/2009 | the United States | USA | 2009.6 | HAdV-C1 | OR753111 | OR753111 | OR753111 | Clade 2 |
| 48 | HAdV-C1/USA/7G10/2011 | the United States | USA | 2011.1 | HAdV-C1 | OR753112 | OR753112 | OR753112 | Clade 2 |
| 49 | HAdV-C1/USA/9J8/2012 | the United States | USA | 2012.4 | HAdV-C1 | OR753113 | OR753113 | OR753113 | Clade 1 |
| 50 | HAdV-C1/USA/7L2/2011 | the United States | USA | 2011.2 | HAdV-C1 | OR753115 | OR753115 | OR753115 | Clade 1 |
| 51 | HAdV-C1/USA/3A8/2009 | the United States | USA | 2009.1 | HAdV-C1 | OR753125 | OR753125 | OR753125 | Clade 2 |
| 52 | HAdV-C1/USA/6E7/2010 | the United States | USA | 2010.4 | HAdV-C1 | OR753128 | OR753128 | OR753128 | Clade 1 |
| 53 | HAdV-C1/USA/5P8/2010 | the United States | USA | 2010.1 | HAdV-C1 | OR753129 | OR753129 | OR753129 | Clade 2 |
| 54 | HAdV-C1/USA/2R7/2009 | the United States | USA | 2009.6 | HAdV-C1 | OR753130 | OR753130 | OR753130 | Clade 1 |
| 55 | HAdV-C1/USA/9D7/2012 | the United States | USA | 2012.3 | HAdV-C1 | OR753132 | OR753132 | OR753132 | Clade 2 |
| 56 | HAdV-C1/USA/8I7/2011 | the United States | USA | 2011.8 | HAdV-C1 | OR753138 | OR753138 | OR753138 | Clade 2 |
| 57 | HAdV-C1/USA/2I6/2019 | the United States | USA | 2019.11 | HAdV-C1 | OR777163 | OR777163 | OR777163 | Clade 1 |
| 58 | HAdV-C1/USA/2I7/2019 | the United States | USA | 2019.11 | HAdV-C1 | OR777164 | OR777164 | OR777164 | Clade 2 |
| 59 | HAdV-C1/USA/2P8/2020 | the United States | USA | 2020.1 | HAdV-C1 | OR777165 | OR777165 | OR777165 | Clade 1 |
| 60 | HAdV-C1/USA/6S8/2010 | the United States | USA | 2010.1 | HAdV-C1 | OR777172 | OR777172 | OR777172 | Clade 2 |
| 61 | HAdV-C1/USA/10F7/2012 | the United States | USA | 2012.12 | HAdV-C1 | OR777176 | OR777176 | OR777176 | Clade 1 |
| 62 | HAdV-C1/USA/10J4/2013 | the United States | USA | 2013.2 | HAdV-C1 | OR777177 | OR777177 | OR777177 | 其他 |
| 63 | HAdV-C1/USA/10P10/2013 | the United States | USA | 2013.6 | HAdV-C1 | OR777178 | OR777178 | OR777178 | Clade 1 |
| 64 | HAdV-C1/USA/12D7/2015 | the United States | USA | 2015.4 | HAdV-C1 | OR777182 | OR777182 | OR777182 | Clade 2 |
| 65 | HAdV-C1/USA/12K10/2015 | the United States | USA | 2015.9 | HAdV-C1 | OR777192 | OR777192 | OR777192 | Clade 1 |
| 66 | HAdV-C1/USA/12L3/2015 | the United States | USA | 2015.9 | HAdV-C1 | OR777194 | OR777194 | OR777194 | Clade 1 |
| 67 | HAdV-C1/USA/13K6/2017 | the United States | USA | 2017.1 | HAdV-C1 | OR777214 | OR777214 | OR777214 | Clade 2 |
| 68 | HAdV-C1/USA/13L9/2017 | the United States | USA | 2017.1 | HAdV-C1 | OR777216 | OR777216 | OR777216 | Clade 2 |
| 69 | HAdV-C1/USA/13R2/2017 | the United States | USA | 2017.6 | HAdV-C1 | OR777221 | OR777221 | OR777221 | Clade 2 |
| 70 | HAdV-C1/USA/13R9/2017 | the United States | USA | 2017.7 | HAdV-C1 | OR777223 | OR777223 | OR777223 | Clade 2 |
| 71 | HAdV-C1/USA/14A1/2017 | the United States | USA | 2017.9 | HAdV-C1 | OR777225 | OR777225 | OR777225 | Clade 2 |
| 72 | HAdV-C1/USA/15E7/2018 | the United States | USA | 2018.4 | HAdV-C1 | OR777231 | OR777231 | OR777231 | Clade 2 |
| 73 | Shenzhen-2023-4-SARI-P1380 | China Shenzhen | CHN/Shenzhen | 2023.4 | HAdV-C1 | √ | √ | √ | Clade 2 |
| 74 | Shenzhen-2023-3-ILI-P145 | China Shenzhen | CHN/Shenzhen | 2023.3 | HAdV-C1 | √ | √ | √ | Clade 2 |
| 75 | Shenzhen-2023-3-ILI-P1464 | China Shenzhen | CHN/Shenzhen | 2023.3 | HAdV-C1 | √ | √ | √ | Clade 2 |
| 76 | Shenzhen-2023-4-ILI-P1775 | China Shenzhen | CHN/Shenzhen | 2023.4 | HAdV-C1 | √ | √ | √ | Clade 2 |
| 77 | Shenzhen-2023-4-ILI-P1778 | China Shenzhen | CHN/Shenzhen | 2023.4 | HAdV-C1 | √ | √ | √ | Clade 2 |
| 78 | Shenzhen-2023-2-ILI-P600 | China Shenzhen | CHN/Shenzhen | 2023.2 | HAdV-C1 | √ | √ | √ | Clade 1 |

**Supplement Table 3.** The nucleotide and amino acid identities of HAdV-C1 and HAdV-B3 strains in this study and other strains in China and other countries

| Gene type | Clade | Strains in our study | Other strains within the same Clade | Similarity | Penton base | Hexon | Fiber |
| --- | --- | --- | --- | --- | --- | --- | --- |
| HAdV-C1 | Clade 1 | Shenzhen-2023-2-ILI-P600 | China | Nucleotide | 99.2-100.0 | 99.7-99.9 | － |
|  |  |  | Other countries |  | 99.0-99.9 | 99.6-100.0 | 99.3-99.9 |
|  |  |  | China | Amino acid | 99.3-100.0 | 99.9-100.0 | － |
|  |  |  | Other countries |  | 98.9-100.0 | 99.9-100.0 | 98.6-99.8 |
|  | Clade 2 | Shenzhen-2023-4-SARI-P1380、Shenzhen-2023-3-ILI-P145、Shenzhen-2023-3-ILI-P1464、Shenzhen-2023-4-ILI-P1775、Shenzhen-2023-4-ILI-P1778 | China | Nucleotide | 99.2-100.0 | 99.7-99.9 | 99.3-99.9 |
|  |  |  | Other countries |  | 99.0-99.9 | 99.6-100.0 | 99.3-99.9 |
|  |  |  | China | Amino acid | 99.3-100.0 | 99.9-100.0 | 99.7-100.0 |
|  |  |  | Other countries |  | 98.9-100.0 | 99.9-100.0 | 99.3-100.0 |
| HAdV-B3 | Clade 2 | 30 strains (Supplement Table 5) | China | Nucleotide | 99.8-100.0 | 98.6-100.0 | 99.8-100.0 |
|  |  |  | Other countries |  | 99.9-100.0 | 98.6-100.0 | 98.3-100.0 |
|  |  |  | China | Amino acid | 99.5-100.0 | 97.4-100.0 | 99.4-100.0 |
|  |  |  | Other countries |  | 99.8-100.0 | 97.4-100.0 | 96.5-100.0 |

**Supplement Table 4.** The average nucleotide p-distance based on three genes of HAdV-C1 and HAdV-B3 strains

| Average nucleotide p-distance | HAdV-C1 | | | HAdV-B3 | | |
| --- | --- | --- | --- | --- | --- | --- |
|  | Penton base | Hexon | Fiber | Penton base | Hexon | Fiber |
| Within Clade 1 | 0.003 | 0.002 | 0.004 | 0.002 | 0.003 | 0.002 |
| Within Clade 2 | － | － | 0.003 | 0.001 | 0.0002 | 0.0008 |
| Between Clade 1 and Clade 2 | － | － | 0.008 | 0.005 | 0.004 | 0.003 |

**Supplement Table 5.** Amino acid site variation of Fiber gene of global HAdV-C1 strains

| Strain name | Clade | Fiber | | | | | | | | | | | | | | | | | | | | | | | | | | | | | | | | | | | | |
| --- | --- | --- | --- | --- | --- | --- | --- | --- | --- | --- | --- | --- | --- | --- | --- | --- | --- | --- | --- | --- | --- | --- | --- | --- | --- | --- | --- | --- | --- | --- | --- | --- | --- | --- | --- | --- | --- | --- |
|  |  | 10 | 22 | 53 | 56 | 63 | 68 | 71 | 74 | 139 | 179 | 184 | 190 | 194 | 199 | 207 | 247 | 259 | 285 | 323 | 334 | 339 | 350 | 375 | 414 | 432 | 442 | 470 | 472 | 490 | 510 | 527 | 544 | 561 | 563 | 565 | 570 | 582 |
| ARG-2000-C1-JX173078 | Clade 1 | T | G | E | V | A | S | A | E | D | P | T | V | N | S | V | T | V | K | L | D | H | T | P | H | V | R | H | S | A | R | E | T | N | T | I | A | R |
| CAN-2016-C1-OM112294 | Clade 1 | . | . | . | . | . | . | . | . | . | . | . | . | . | . | . | . | . | . | . | . | . | . | . | . | . | K | . | . | . | . | D | . | . | . | . | . | G |
| SG-2016-C1-MN513341 (2 strains) | Clade 1 | . | D | . | . | . | . | . | . | . | . | . | . | . | . | . | . | . | . | . | . | . | . | . | . | . | . | . | . | . | . | . | . | . | . | . | . | . |
| SG-2016-C1-MN513344 | Clade 1 | . | . | . | . | . | . | . | . | . | . | . | . | . | . | . | . | . | . | . | N | . | . | . | Y | . | K | . | . | . | . | . | . | T | . | M | . | G |
| USA-1953-C1-AF534906 | Clade 1 | . | . | . | . | . | . | . | K | . | . | . | . | . | N | . | . | . | . | . | . | . | . | . | . | . | . | . | . | . | . | . | . | . | . | . | . | . |
| USA-2003-C1-JX173082 | Clade 1 | . | . | . | . | . | . | . | . | . | . | . | . | . | N | . | . | . | . | . | . | . | . | . | . | . | . | . | . | . | . | . | . | . | . | . | . | . |
| USA-2009-C1-OQ518300 (4 strains) | Clade 1 | . | . | . | . | . | . | . | . | . | . | . | . | . | N | . | . | . | . | . | . | . | . | . | . | . | . | . | . | . | . | . | . | . | . | . | . | . |
| USA-2009-C1-OR753130 | Clade 1 | . | . | . | . | . | . | . | . | . | . | . | . | . | . | . | . | . | . | . | . | . | . | . | . | . | K | . | . | . | . | D | . | . | . | . | . | G |
| USA-2010-C1-OQ518269 | Clade 1 | . | . | . | . | . | N | . | . | . | . | . | . | . | N | . | . | . | . | . | . | . | . | . | . | . | . | . | . | T | . | . | . | . | . | . | . | . |
| USA-2010-C1-OR753096 | Clade 1 | . | . | . | . | . | . | . | . | . | . | . | . | . | . | . | . | . | . | . | . | . | . | . | . | . | . | . | . | . | . | . | . | . | . | . | . | . |
| USA-2010-C1-OR753128 (2 strains) | Clade 1 | . | . | . | . | . | . | . | . | . | . | . | . | . | . | . | . | . | . | . | . | . | . | . | . | . | . | . | . | . | . | . | . | . | . | . | . | . |
| USA-2011-C1-OQ518259 (3 strains) | Clade 1 | . | . | . | . | . | . | . | . | . | . | . | . | . | N | . | . | . | . | . | . | . | . | . | . | . | . | . | . | . | . | . | . | . | . | . | . | . |
| USA-2011-C1-OR735184 | Clade 1 | . | . | . | . | . | . | . | . | . | . | . | . | . | . | . | A | . | . | . | . | . | . | . | . | . | . | . | . | . | . | . | . | . | . | . | T | . |
| USA-2011-C1-OR735206 | Clade 1 | . | . | . | . | . | . | . | . | . | . | . | . | . | . | . | . | . | . | . | . | . | S | . | . | . | K | . | . | . | . | . | . | . | . | . | . | G |
| USA-2011-C1-OR753115 | Clade 1 | . | . | . | . | . | . | . | . | . | . | . | . | . | . | . | . | . | . | . | . | . | . | . | . | . | K | . | . | . | . | D | . | . | . | . | . | G |
| USA-2012-C1-OQ518338 | Clade 1 | . | . | . | . | . | . | . | . | . | . | . | . | . | N | . | . | . | . | . | . | . | . | . | . | . | . | . | . | . | . | . | . | . | . | . | . | . |
| USA-2012-C1-OR735190 | Clade 1 | . | . | . | . | . | . | . | . | N | . | . | . | D | . | . | . | . | . | . | . | . | . | . | . | . | K | . | . | . | . | . | . | . | . | . | . | G |
| USA-2012-C1-OR753113 | Clade 1 | . | . | . | . | . | . | . | . | . | . | . | . | . | . | . | . | . | . | . | . | . | . | . | Y | . | K | . | . | . | . | . | . | . | S | M | . | G |
| USA-2012-C1-OR777176 | Clade 1 | . | . | . | . | . | . | . | . | . | . | . | . | . | . | . | . | . | . | . | . | . | . | . | . | . | . | . | . | . | . | . | . | . | . | . | . | . |
| USA-2013-C1-OR735198 | Clade 1 | . | . | . | . | . | . | . | . | . | S | . | . | . | . | . | . | . | . | . | . | . | . | . | . | . | . | . | . | . | . | . | . | . | . | . | . | . |
| USA-2013-C1-OR735199 | Clade 1 | . | . | . | . | . | . | . | . | . | . | . | . | . | . | . | . | . | . | . | . | . | . | . | . | . | . | . | . | . | . | . | . | . | . | . | . | . |
| USA-2013-C1-OR777178 | Clade 1 | . | . | . | . | . | . | . | . | . | . | . | . | . | . | . | . | . | . | . | . | . | . | . | . | . | K | . | . | . | . | . | . | . | . | . | . | G |
| USA-2015-C1-OR777192 | Clade 1 | . | . | . | . | . | . | . | . | . | . | . | . | . | . | . | A | . | . | I | . | . | . | . | . | . | . | . | . | . | . | . | . | . | . | . | . | . |
| USA-2015-C1-OR777194 | Clade 1 | . | . | . | . | . | . | . | . | N | . | . | . | D | . | . | . | . | . | . | . | . | . | . | . | . | K | . | . | . | . | . | . | . | . | . | . | G |
| USA-2019-C1-OR777163 (2 strains) | Clade 1 | . | . | . | . | . | . | . | . | . | . | . | . | . | . | I | . | . | . | . | . | . | . | . | . | . | . | . | . | . | . | . | . | . | . | . | . | . |
| USA-2020-C1-OR777165 | Clade 1 | . | . | . | . | . | . | . | . | . | . | . | . | . | N | . | . | . | . | . | . | . | . | . | . | . | . | . | . | . | . | . | . | . | . | . | . | . |
| Shenzhen-2023-2-ILI-P600 | Clade 1 | . | . | . | . | . | . | . | . | . | . | . | . | . | . | . | . | . | . | . | . | . | . | . | Y | . | K | . | . | . | . | . | . | T | . | M | . | G |
| CAN-2015-C1-OM112288 | Clade 2 | . | . | . | . | . | . | . | . | . | . | . | . | . | . | . | . | . | . | . | . | N | . | . | Y | . | K | . | G | . | . | D | . | . | . | . | . | G |
| CAN-2015-C1-OM112292 | Clade 2 | . | . | . | . | . | . | T | . | . | . | . | I | . | . | . | . | . | . | . | . | N | . | . | Y | . | K | . | G | . | . | D | . | . | . | . | . | G |
| CAN-2015-C1-OM112293 | Clade 2 | . | . | . | . | . | . | . | . | . | . | . | . | . | . | . | . | . | . | . | . | N | . | . | Y | . | K | . | G | . | . | D | . | . | . | . | . | G |
| CAN-2018-C1-OM112290 | Clade 2 | . | . | . | . | . | . | . | . | . | . | . | . | . | . | . | . | . | . | . | . | N | . | . | Y | . | K | . | G | . | . | D | . | . | . | . | . | G |
| CHN/Shanghai-2016-C1-MH183293 | Clade 2 | . | . | . | . | . | . | T | . | . | . | . | . | . | . | . | . | . | . | . | . | N | . | . | Y | I | K | N | G | . | . | D | . | . | . | . | . | G |
| CHN/Qinghai-2018-C1-MN737436 | Clade 2 | . | . | . | . | . | . | T | . | . | . | . | . | . | . | . | . | . | . | . | . | N | . | . | Y | . | K | . | G | . | . | D | . | . | . | . | . | G |
| EGY-2001-C1-JX173080 | Clade 2 | . | . | . | . | . | . | T | . | . | . | . | . | . | . | . | . | . | . | . | . | N | . | . | Y | I | K | N | G | . | . | D | . | . | . | . | . | G |
| USA-1968-C1-OQ518270 | Clade 2 | . | . | . | . | . | . | . | . | . | . | . | . | . | . | . | . | M | . | . | . | N | . | . | Y | . | K | . | G | . | . | D | . | . | . | . | . | G |
| USA-2003-C1-JX173083 | Clade 2 | . | . | . | . | . | . | . | . | . | . | . | . | . | . | . | . | . | . | . | . | N | . | . | Y | . | K | . | G | . | . | D | . | . | . | . | . | G |
| USA-2003-C1-JX173085 | Clade 2 | . | . | . | . | . | . | . | . | . | . | . | . | . | . | . | . | . | . | . | . | N | . | . | Y | . | K | . | G | . | . | D | . | . | . | . | . | G |
| USA-2004-C1-JX173086 | Clade 2 | . | . | . | . | . | . | . | . | . | . | . | . | . | . | . | . | . | . | . | . | N | . | . | Y | . | K | . | G | . | . | D | . | . | . | . | . | G |
| USA-2009-C1-OQ518262 (2 strains) | Clade 2 | . | . | . | . | . | . | T | . | . | . | . | . | . | . | . | . | . | . | . | . | N | . | . | Y | . | K | . | G | . | . | D | . | . | . | . | . | G |
| USA-2009-C1-OQ518311 | Clade 2 | . | . | . | . | . | . | . | . | . | . | . | . | . | . | . | . | . | . | . | . | N | . | . | Y | . | K | . | G | . | . | D | . | . | . | . | . | G |
| USA-2010-C1-OR753129 | Clade 2 | . | . | . | . | . | . | . | . | . | . | . | . | . | . | . | . | . | . | . | . | N | . | . | Y | . | K | . | G | . | . | D | . | . | . | . | . | G |
| USA-2010-C1-OR777172 | Clade 2 | . | . | . | . | . | . | . | . | . | . | . | . | . | . | . | . | . | . | . | . | N | . | . | Y | . | K | . | G | . | . | D | . | . | . | . | . | G |
| USA-2011-C1-OQ518310 | Clade 2 | . | . | . | . | . | . | T | . | . | . | . | . | . | . | . | . | . | . | . | . | N | . | . | Y | . | K | . | G | . | . | D | . | . | . | . | . | G |
| USA-2011-C1-OR735181 (2 strains) | Clade 2 | . | . | . | . | . | . | . | . | . | . | . | . | . | . | . | . | . | . | . | . | N | . | . | Y | . | K | . | G | . | . | D | . | . | . | . | . | G |
| USA-2011-C1-OR735194 | Clade 2 | . | . | . | . | . | . | . | . | . | . | . | I | . | . | . | . | . | . | . | . | N | . | . | Y | . | K | . | G | . | . | D | . | . | . | . | . | G |
| USA-2011-C1-OR753138 | Clade 2 | . | . | . | . | . | . | . | . | . | . | . | . | . | . | . | . | . | . | . | . | N | . | . | Y | . | K | . | G | . | . | D | . | . | . | . | . | G |
| USA-2012-C1-OR735200 | Clade 2 | A | . | . | . | . | . | . | . | . | . | . | . | . | . | . | . | . | . | . | . | N | . | . | Y | . | K | . | G | . | . | D | . | . | . | . | . | G |
| USA-2012-C1-OR753132 (5 strains) | Clade 2 | . | . | . | . | . | . | . | . | . | . | . | . | . | . | . | . | . | . | . | . | N | . | . | Y | . | K | . | G | . | . | D | . | . | . | . | . | G |
| USA-2013-C1-OQ518346 (3 strains) | Clade 2 | A | . | . | . | . | . | . | . | . | . | . | . | . | . | . | . | . | . | . | . | N | . | . | Y | . | K | . | G | . | . | D | . | . | . | . | . | G |
| USA-2015-C1-OR777182 | Clade 2 | . | . | . | . | . | . | . | . | . | . | . | . | . | . | . | . | . | . | . | . | N | . | . | Y | . | K | . | G | . | . | D | . | . | . | . | . | G |
| USA-2017-C1-OR777223 (5 strains) | Clade 2 | . | . | . | . | . | . | . | . | . | . | . | . | . | . | . | . | . | . | . | . | N | . | . | Y | . | K | . | G | . | . | D | . | . | . | . | . | G |
| USA-2018-C1-OR777231 | Clade 2 | . | . | . | . | . | . | T | . | . | . | . | . | . | . | . | . | . | . | . | . | N | . | . | Y | . | K | . | G | . | . | D | . | . | . | . | . | G |
| Shenzhen-2023-4-ILI-P1778 | Clade 2 | . | . | . | . | . | . | T | . | . | . | . | . | . | . | . | . | . | . | . | . | N | . | . | Y | I | K | N | G | . | . | D | . | . | . | . | . | G |
| Shenzhen-2023-4-ILI-P1775 | Clade 2 | . | . | . | . | . | . | T | . | . | . | . | . | . | . | . | . | . | . | . | . | N | . | . | Y | I | K | N | G | . | . | D | . | . | . | . | . | G |
| Shenzhen-2023-3-ILI-P1464 | Clade 2 | . | . | . | . | . | . | T | . | . | . | . | . | . | . | . | . | . | . | . | . | N | . | . | Y | I | K | N | G | . | . | D | . | . | . | . | . | G |
| Shenzhen-2023-3-ILI-P1457 | Clade 2 | . | . | . | . | . | . | T | . | . | . | . | . | . | . | . | . | . | . | . | . | N | . | . | Y | I | K | N | G | . | . | D | . | . | . | . | . | G |
| Shenzhen-2023-4-SARI-P1380 | Clade 2 | . | . | . | . | . | . | T | . | . | . | . | . | . | . | . | . | . | . | . | . | N | . | . | Y | I | K | N | G | . | . | D | . | . | . | . | . | G |

**Supplement Table 6.** The sequence information of global HAdV-B3 strains

| No. | Strain Name in GenBank | Country/Province | Regional abbreviation | Collection Date | Genotype | Accession Number | | | Clade | | |
| --- | --- | --- | --- | --- | --- | --- | --- | --- | --- | --- | --- |
|  |  |  |  |  |  | Penton base | Hexon | Fiber | Penton base | Hexon | Fiber |
| 1 | BJ02/CHN/2011 | China Beijing | CHN/Beijing | 2011 | HAdV-B3 | KP270907 | KM458623 | KP270916 | Clade 2 | Clade 2 | Clade 2 |
| 2 | BJ03/CHN/2012 | China Beijing | CHN/Beijing | 2012 | HAdV-B3 | KP270908 | KM458624 | KP270917 | Clade 2 | Clade 2 | Clade 2 |
| 3 | BJ19/CHN/2013 | China Beijing | CHN/Beijing | 2013 | HAdV-B3 | KP270914 | KM458630 | KP270922 | Clade 2 | Clade 2 | Clade 2 |
| 4 | BJ20170382 | China Beijing | CHN/Beijing | 2017 | HAdV-B3 | MW748640 | MW748618 | MW748596 | Clade 2 | Clade 2 | Clade 2 |
| 5 | BJ20170380 | China Beijing | CHN/Beijing | 2017 | HAdV-B3 | MW748639 | MW748617 | MW748595 | Clade 2 | Clade 2 | Clade 2 |
| 6 | BJ20170371 | China Beijing | CHN/Beijing | 2017 | HAdV-B3 | MW748638 | MW748616 | MW748594 | Clade 2 | Clade 2 | Clade 2 |
| 7 | BJ20170365 | China Beijing | CHN/Beijing | 2017 | HAdV-B3 | MW748637 | MW748615 | MW748593 | Clade 2 | Clade 2 | Clade 2 |
| 8 | BJ20170306 | China Beijing | CHN/Beijing | 2017 | HAdV-B3 | MW748636 | MW748614 | MW748592 | Clade 2 | Clade 2 | Clade 2 |
| 9 | BJ20170268 | China Beijing | CHN/Beijing | 2017 | HAdV-B3 | MW748635 | MW748613 | MW748591 | Clade 2 | Clade 2 | Clade 2 |
| 10 | BJ20170262 | China Beijing | CHN/Beijing | 2017 | HAdV-B3 | MW748634 | MW748612 | MW748590 | Clade 2 | Clade 2 | Clade 2 |
| 11 | BJ20170260 | China Beijing | CHN/Beijing | 2017 | HAdV-B3 | MW748633 | MW748611 | MW748589 | Clade 2 | Clade 2 | Clade 2 |
| 12 | BJ20170246 | China Beijing | CHN/Beijing | 2017 | HAdV-B3 | MW748632 | MW748610 | MW748588 | Clade 2 | Clade 2 | Clade 2 |
| 13 | HB20150330 | China Beijing | CHN/Beijing | 2015 | HAdV-B3 | MW748631 | MW748609 | MW748587 | Clade 2 | Clade 2 | Clade 2 |
| 14 | HB20150116 | China Beijing | CHN/Beijing | 2015 | HAdV-B3 | MW748630 | MW748608 | MW748586 | Clade 2 | Clade 2 | Clade 2 |
| 15 | WZ20150089 | China Beijing | CHN/Beijing | 2015 | HAdV-B3 | MW748629 | MW748607 | MW748585 | Clade 2 | Clade 2 | Clade 2 |
| 16 | WZ20150088 | China Beijing | CHN/Beijing | 2015 | HAdV-B3 | MW748628 | MW748606 | MW748584 | Clade 2 | Clade 2 | Clade 2 |
| 17 | WZ20150076 | China Beijing | CHN/Beijing | 2015 | HAdV-B3 | MW748627 | MW748605 | MW748583 | Clade 2 | Clade 2 | Clade 2 |
| 18 | WZ20150074 | China Beijing | CHN/Beijing | 2015 | HAdV-B3 | MW748626 | MW748604 | MW748582 | Clade 2 | Clade 2 | Clade 2 |
| 19 | WZ20150072 | China Beijing | CHN/Beijing | 2015 | HAdV-B3 | MW748625 | MW748603 | MW748581 | Clade 2 | Clade 2 | Clade 2 |
| 20 | WZ20150071 | China Beijing | CHN/Beijing | 2015 | HAdV-B3 | MW748624 | MW748602 | MW748580 | Clade 2 | Clade 2 | Clade 2 |
| 21 | SH20160054 | China Beijing | CHN/Beijing | 2016 | HAdV-B3 | MW748623 | MW748601 | MW748579 | Clade 2 | Clade 2 | Clade 2 |
| 22 | SH20160050 | China Beijing | CHN/Beijing | 2016 | HAdV-B3 | MW748622 | MW748600 | MW748578 | Clade 2 | Clade 2 | Clade 2 |
| 23 | GZ20150047 | China Beijing | CHN/Beijing | 2015 | HAdV-B3 | MW748621 | MW748599 | MW748577 | Clade 2 | Clade 2 | Clade 2 |
| 24 | GZ20150038 | China Beijing | CHN/Beijing | 2015 | HAdV-B3 | MW748620 | MW748598 | MW748576 | Clade 2 | Clade 2 | Clade 2 |
| 25 | GZ20150033 | China Beijing | CHN/Beijing | 2015 | HAdV-B3 | MW748619 | MW748597 | MW748575 | Clade 2 | Clade 2 | Clade 2 |
| 26 | Guangzhou02 | China Guangdong | CHN/Guangdong | 2004.7 | HAdV-B3 | DQ105654 | DQ105654 | DQ105654 | Clade 2 | Clade 2 | Clade 2 |
| 27 | Guangzhou01 | China Guangdong | CHN/Guangdong | 2005.1 | HAdV-B3 | DQ099432 | DQ099432 | DQ099432 | Clade 2 | Clade 2 | Clade 2 |
| 28 | GZ13 | China Guangdong | CHN/Guangdong | 2011.1 | HAdV-B3 | - | JQ764730 | - | - | Clade 2 | - |
| 29 | HZ121 | China Hangzhou | CHN/Hangzhou | 2011.3 | HAdV-B3 | - | KF551934 | - | - | Clade 2 | - |
| 30 | Hangzhou-2011 | China Hangzhou | CHN/Hangzhou | 2011.5 | HAdV-B3 | - | KF511679 | - | - | Other | - |
| 31 | HK42 | China Hong Kang | CHN/Hong Kang | 2014.8 | HAdV-B3 | - | - | OM988094 | - | - | Clade 2 |
| 32 | Human/China/Tongliao/2019/6[P3H3F3] | China Inner Mongolia | CHN/Inner Mongolia | 2019.6 | HAdV-B3 | MW767985 | MW767985 | MW767985 | Clade 2 | Clade 2 | Clade 2 |
| 33 | Human/China/Shanghai/3496/3[P3H3F3]/2009 | China Shanghai | CHN/Shanghai | 2011.8 | HAdV-B3 | MK847517 | MK847517 | MK847517 | Clade 2 | Clade 2 | Clade 2 |
| 34 | Human/China/Shanghai/4010/2012/3[P3H3F3] | China Shanghai | CHN/Shanghai | 2012.3 | HAdV-B3 | MK883608 | MK883608 | MK883608 | Clade 2 | Clade 2 | Clade 2 |
| 35 | Human/China/Shanghai/3754/2011/3[P3H3F3] | China Shanghai | CHN/Shanghai | 2011.1 | HAdV-B3 | MK883604 | MK883604 | MK883604 | Clade 2 | Clade 2 | Clade 2 |
| 36 | Human/China/Shanghai/3517/2011/3[P3H3F3] | China Shanghai | CHN/Shanghai | 2011.8 | HAdV-B3 | MK883603 | MK883603 | MK883603 | Clade 2 | Clade 2 | Clade 2 |
| 37 | Human/China/Shanghai/3096/2009/3[P3H3F3] | China Shanghai | CHN/Shanghai | 2009.12 | HAdV-B3 | MK836311 | MK836311 | MK836311 | Clade 2 | Clade 2 | Clade 2 |
| 38 | human/China/Shanghai/1631/2009/3[P3H3F3] | China Shanghai | CHN/Shanghai | 2009.1 | HAdV-B3 | MK836310 | MK836310 | MK836310 | Clade 2 | Clade 2 | Clade 2 |
| 39 | human/China/Shanghai/678/2009/3[P3H3F3] | China Shanghai | CHN/Shanghai | 2009.6 | HAdV-B3 | MK836308 | MK836308 | MK836308 | Clade 2 | Clade 2 | Clade 2 |
| 40 | human/China/Shanghai/538/2009/3[P3H3F3] | China Shanghai | CHN/Shanghai | 2009.6 | HAdV-B3 | MK813915 | MK813915 | MK813915 | Clade 2 | Clade 2 | Clade 2 |
| 41 | human/China/Shanghai/381/2004/3[P3H3F3] | China Shanghai | CHN/Shanghai | 2009.6 | HAdV-B3 | MK813914 | MK813914 | MK813914 | Clade 2 | Clade 2 | Clade 2 |
| 42 | Shanxi2019-15 | China Shanxi | CHN/Shanxi | 2019 | HAdV-B3 | OQ128168 | OQ128162 | OQ128152 | Clade 2 | Clade 2 | Clade 2 |
| 43 | Shanxi2018-44 | China Shanxi | CHN/Shanxi | 2018 | HAdV-B3 | OQ128167 | OQ128161 | OQ128151 | Clade 2 | Clade 2 | Clade 2 |
| 44 | Shanxi2018-38 | China Shanxi | CHN/Shanxi | 2018 | HAdV-B3 | OQ128172 | OQ128160 | OQ128150 | Clade 2 | Clade 2 | Clade 2 |
| 45 | Shanxi2018-37 | China Shanxi | CHN/Shanxi | 2018 | HAdV-B3 | OQ128171 | OQ128159 | OQ128149 | Clade 2 | Clade 2 | Clade 2 |
| 46 | Shanxi2018-26 | China Shanxi | CHN/Shanxi | 2018 | HAdV-B3 | OQ128166 | OQ128158 | OQ128148 | Clade 2 | Clade 2 | Clade 2 |
| 47 | Shanxi2018-25 | China Shanxi | CHN/Shanxi | 2018 | HAdV-B3 | OQ128165 | OQ128157 | OQ128147 | Clade 2 | Clade 2 | Clade 2 |
| 48 | Shanxi2018-24 | China Shanxi | CHN/Shanxi | 2018 | HAdV-B3 | OQ128170 | OQ128156 | OQ128146 | Clade 2 | Clade 2 | Clade 2 |
| 49 | Shanxi2018-15 | China Shanxi | CHN/Shanxi | 2018 | HAdV-B3 | OQ128164 | OQ128155 | OQ128145 | Clade 2 | Clade 2 | Clade 2 |
| 50 | Shanxi2018-5 | China Shanxi | CHN/Shanxi | 2018 | HAdV-B3 | OQ128163 | OQ128154 | OQ128144 | Clade 2 | Clade 2 | Clade 2 |
| 51 | Shanxi2018-3 | China Shanxi | CHN/Shanxi | 2018 | HAdV-B3 | OQ128169 | OQ128153 | OQ128143 | Clade 2 | Clade 2 | Clade 2 |
| 52 | 1460-04-TW | China Taiwan | CHN/Taiwan | 2004 | HAdV-B3 | - | EF494643 | - | - | Clade 1 | - |
| 53 | 1964-99-TW | China Taiwan | CHN/Taiwan | 1964 | HAdV-B3 | - | EF494642 | - | - | Clade 2 | - |
| 54 | 0198Taiwan2011 | China Taiwan | CHN/Taiwan | 2011 | HAdV-B3 | - | KC456084 | KC456116 | - | Clade 2 | Clade 2 |
| 55 | 0456Taiwan2011 | China Taiwan | CHN/Taiwan | 2011 | HAdV-B3 | - | KC456085 | KC456122 | - | Clade 2 | Clade 2 |
| 56 | 0552Taiwan2011 | China Taiwan | CHN/Taiwan | 2011 | HAdV-B3 | - | KC456086 | KC456123 | - | Clade 2 | Clade 2 |
| 57 | 0736Taiwan2011 | China Taiwan | CHN/Taiwan | 2011 | HAdV-B3 | - | KC456087 | KC456124 | - | Clade 2 | Clade 2 |
| 58 | 0889Taiwan2011 | China Taiwan | CHN/Taiwan | 2011 | HAdV-B3 | - | KC456088 | KC456125 | - | Clade 2 | Clade 2 |
| 59 | 1012Taiwan2011 | China Taiwan | CHN/Taiwan | 2011 | HAdV-B3 | - | KC456089 | KC456105 | - | Clade 2 | Clade 2 |
| 60 | 1112Taiwan2011 | China Taiwan | CHN/Taiwan | 2011 | HAdV-B3 | - | KC456090 | KC456106 | - | Clade 2 | Clade 2 |
| 61 | 1133Taiwan2011 | China Taiwan | CHN/Taiwan | 2011 | HAdV-B3 | - | KC456091 | KC456108 | - | Clade 2 | Clade 2 |
| 62 | 1225Taiwan2011 | China Taiwan | CHN/Taiwan | 2011 | HAdV-B3 | - | KC456092 | KC456104 | - | Clade 2 | Clade 2 |
| 63 | 1301Taiwan2011 | China Taiwan | CHN/Taiwan | 2011 | HAdV-B3 | - | KC456093 | KC456107 | - | Clade 2 | Clade 2 |
| 64 | 1332Taiwan2011 | China Taiwan | CHN/Taiwan | 2011 | HAdV-B3 | - | KC456094 | KC456109 | - | Clade 2 | Clade 2 |
| 65 | 1444Taiwan2011 | China Taiwan | CHN/Taiwan | 2011 | HAdV-B3 | - | KC456095 | KC456110 | - | Clade 2 | Clade 2 |
| 66 | 1697Taiwan2011 | China Taiwan | CHN/Taiwan | 2011 | HAdV-B3 | - | KC456096 | KC456111 | - | Clade 2 | Clade 2 |
| 67 | 1735Taiwan2011 | China Taiwan | CHN/Taiwan | 2011 | HAdV-B3 | - | KC456097 | KC456112 | - | Clade 2 | Clade 2 |
| 68 | 1764Taiwan2011 | China Taiwan | CHN/Taiwan | 2011 | HAdV-B3 | - | KC456098 | KC456113 | - | Clade 2 | Clade 2 |
| 69 | 1854Taiwan2011 | China Taiwan | CHN/Taiwan | 2011 | HAdV-B3 | - | KC456099 | KC456114 | - | Clade 2 | Clade 2 |
| 70 | 1962Taiwan2011 | China Taiwan | CHN/Taiwan | 2011 | HAdV-B3 | - | KC456100 | KC456115 | - | Clade 2 | Clade 2 |
| 71 | 2902Taiwan2011 | China Taiwan | CHN/Taiwan | 2011 | HAdV-B3 | - | KC456083 | KC456117 | - | Clade 2 | Clade 2 |
| 72 | 3234Taiwan2011 | China Taiwan | CHN/Taiwan | 2011 | HAdV-B3 | - | KC456101 | KC456118 | - | Clade 2 | Clade 2 |
| 73 | 3440Taiwan2011 | China Taiwan | CHN/Taiwan | 2011 | HAdV-B3 | - | KC456102 | KC456119 | - | Clade 2 | Clade 2 |
| 74 | 3759Taiwan2011 | China Taiwan | CHN/Taiwan | 2011 | HAdV-B3 | - | KC456103 | KC456120 | - | Clade 2 | Clade 2 |
| 75 | 0424/Taiwan/2011 | China Taiwan | CHN/Taiwan | 2011 | HAdV-B3 | - | - | KC456121 | - | - | Clade 2 |
| 76 | human/IND/MEEI_00062/2011/3[P7H3F3] | India | IND | 2011.4 | HAdV-B3 | - | KF268212 | KF268212 | - | Clade 2 | Clade 2 |
| 77 | human/IND/MEEI_00057/2011/3[P7H3F3] | India | IND | 2011.4 | HAdV-B3 | - | KF268210 | KF268210 | - | Clade 1 | Other |
| 78 | human/Japan/F1_Adv3a52/1988 | Japan | JPN | 1988 | HAdV-B3 | AB900147 | AB900148 | AB900149 | Clade 1 | Clade 1 | Clade 1 |
| 79 | human/Japan/F23_Adv3a53/2003 | Japan | JPN | 2003 | HAdV-B3 | AB900150 | AB900151 | AB900152 | Clade 2 | Clade 2 | Clade 2 |
| 80 | human/Japan/F30_Adv3a54/2004 | Japan | JPN | 2004 | HAdV-B3 | AB900153 | AB900154 | AB900155 | Clade 2 | Clade 2 | Clade 2 |
| 81 | human/JPN/TKYAd191191/2019/3 | Japan | JPN | 2019.8 | HAdV-B3 | LC695001 | LC695001 | LC695001 | Clade 1 | Clade 1 | Clade 1 |
| 82 | KNIH Ad00/17 | Korea | KOR | 2003 | HAdV-B3 | - | AF542126 | AY224416 | - | Other | Clade 2 |
| 83 | KUMC-62 | Korea | KOR | 2015 | HAdV-B3 | KY320276 | KY320276 | KY320276 | Clade 2 | Clade 2 | Clade 2 |
| 84 | GB（prototype) | the United States | USA | 1953 | HAdV-B3 | AY599834 | AY599834 | AY599834 | Other | Other | Other |
| 85 | T382/Ft Jackson South Carolina USA/2002 | the United States | USA | 2002 | HAdV-B3 | KX384958 | KX384958 | KX384958 | Clade 2 | Clade 2 | Clade 2 |
| 86 | human/USA/MEEI_00075/X/3[P3H3F3] | the United States | USA | 2003 | HAdV-B3 | KF268202 | KF268202 | KF268202 | Clade 1 | Clade 1 | Clade 1 |
| 87 | human/USA/CL_45/1988/3[P3H7F3] | the United States | USA | 1988 | HAdV-B3 | KF268132 | - | KF268132 | Clade 2 | - | Clade 2 |
| 88 | human/USA/Pitts_00112/1988/NEW[P3H3F7] | the United States | USA | 1988 | HAdV-B3 | KF429752 | KF429752 | - | Clade 2 | Clade 2 | - |
| 89 | human/USA/UFL_Adv3/2004/3[P3/H3/F3] | the United States | USA | 2004.1 | HAdV-B3 | KF268195 | KF268195 | KF268195 | Clade 2 | Clade 2 | Clade 2 |
| 90 | HAdV-B3/USA/9N2/2012 | the United States | USA | 2012.7 | HAdV-B3 | PP068614 | PP068614 | PP068614 | Clade 2 | Clade 2 | Clade 2 |
| 91 | HAdV-B3/USA/7D8/2010 | the United States | USA | 2010.11 | HAdV-B3 | OR876397 | OR876397 | OR876397 | Clade 2 | Clade 2 | Clade 2 |
| 92 | HAdV-B3/USA/10Q2/2013 | the United States | USA | 2013.7 | HAdV-B3 | OQ518344 | OQ518344 | OQ518344 | Clade 2 | Clade 2 | Clade 2 |
| 93 | HAdV-B3/USA/5J7/2009 | the United States | USA | 2009.11 | HAdV-B3 | OQ518323 | OQ518323 | OQ518323 | Clade 2 | Clade 2 | Clade 2 |
| 94 | HAdV-B3/USA/10Q1/2013 | the United States | USA | 2013.7 | HAdV-B3 | OQ518322 | OQ518322 | OQ518322 | Clade 2 | Clade 2 | Clade 2 |
| 95 | HAdV-B3/USA/6I2/2010 | the United States | USA | 2010.5 | HAdV-B3 | OQ518321 | OQ518321 | OQ518321 | Clade 2 | Clade 2 | Clade 2 |
| 96 | HAdV-B3/USA/10N6/2013 | the United States | USA | 2013.5 | HAdV-B3 | OQ518317 | OQ518317 | OQ518317 | Clade 2 | Clade 2 | Clade 2 |
| 97 | HAdV-B3/USA/2E9/2009 | the United States | USA | 2009.5 | HAdV-B3 | OQ518315 | OQ518315 | OQ518315 | Clade 2 | Clade 2 | Clade 2 |
| 98 | HAdV-B3/USA/7G3/2011 | the United States | USA | 2011.1 | HAdV-B3 | OQ518306 | OQ518306 | OQ518306 | Clade 2 | Clade 2 | Clade 2 |
| 99 | HAdV-B3/USA/7Q1/2011 | the United States | USA | 2011.3 | HAdV-B3 | OQ518299 | OQ518299 | OQ518299 | Clade 2 | Clade 2 | Clade 2 |
| 100 | HAdV-B3/USA/11D3/2013 | the United States | USA | 2013.11 | HAdV-B3 | OQ518292 | OQ518292 | OQ518292 | Clade 2 | Clade 2 | Clade 2 |
| 101 | HAdV-B3/USA/10N2/2013 | the United States | USA | 2013.5 | HAdV-B3 | OQ518287 | OQ518287 | OQ518287 | Clade 2 | Clade 2 | Clade 2 |
| 102 | HAdV-B3/USA/5K2/2009 | the United States | USA | 2009.11 | HAdV-B3 | OQ518281 | OQ518281 | OQ518281 | Clade 2 | Clade 2 | Clade 2 |
| 103 | HAdV-B3/USA/7K5/2011 | the United States | USA | 2011.2 | HAdV-B3 | OQ518278 | OQ518278 | OQ518278 | Clade 2 | Clade 2 | Clade 2 |
| 104 | HAdV-B3/USA/4R8/2009 | the United States | USA | 2009.11 | HAdV-B3 | OQ518276 | OQ518276 | OQ518276 | Clade 2 | Clade 2 | Clade 2 |
| 105 | HAdV-B3/USA/5K5/2009 | the United States | USA | 2009.12 | HAdV-B3 | OQ518267 | OQ518267 | OQ518267 | Clade 2 | Clade 2 | Clade 2 |
| 106 | HAdV-B3/USA/9A4/2012 | the United States | USA | 2012.1 | HAdV-B3 | OQ518266 | OQ518266 | OQ518266 | Clade 2 | Clade 2 | Clade 2 |
| 107 | HAdV-B3/USA/5S10/2010 | the United States | USA | 2010.2 | HAdV-B3 | OQ518265 | OQ518265 | OQ518265 | Clade 2 | Clade 2 | Clade 2 |
| 108 | HAdV-B3/USA/6Q2/2010 | the United States | USA | 2010.9 | HAdV-B3 | OQ518260 | OQ518260 | OQ518260 | Clade 2 | Clade 2 | Clade 2 |
| 109 | HAdV-B3/USA/16M5/2019 | the United States | USA | 2019.1 | HAdV-B3 | OR777235 | OR777235 | OR777235 | Clade 2 | Clade 2 | Clade 2 |
| 110 | HAdV-B3/USA/12R3/2016 | the United States | USA | 2016.1 | HAdV-B3 | OR777202 | OR777202 | OR777202 | Clade 2 | Clade 2 | Clade 2 |
| 111 | HAdV-B3/USA/12P2/2015 | the United States | USA | 2015.12 | HAdV-B3 | OR777198 | OR777198 | OR777198 | Clade 2 | Clade 2 | Clade 2 |
| 112 | HAdV-B3/USA/12O4/2015 | the United States | USA | 2015.12 | HAdV-B3 | OR777196 | OR777196 | OR777196 | Clade 2 | Clade 2 | Clade 2 |
| 113 | HAdV-B3/USA/9C1/2019 | the United States | USA | 2019.3 | HAdV-B3 | OR777175 | OR777175 | OR777175 | Clade 2 | Clade 2 | Clade 2 |
| 114 | HAdV-B3/USA/6R5/2010 | the United States | USA | 2010.9 | HAdV-B3 | OR777169 | OR777169 | OR777169 | Clade 2 | Clade 2 | Clade 2 |
| 115 | HAdV-B3/USA/6J2/2010 | the United States | USA | 2010.6 | HAdV-B3 | OR777168 | OR777168 | OR777168 | Clade 2 | Clade 2 | Clade 2 |
| 116 | HAdV-B3/USA/1B10/2020 | the United States | USA | 2020.4 | HAdV-B3 | OR777156 | OR777156 | OR777156 | Clade 2 | Clade 2 | Clade 2 |
| 117 | HAdV-B3/USA/8T6/2012 | the United States | USA | 2012.1 | HAdV-B3 | OR753127 | OR753127 | OR753127 | Clade 2 | Clade 2 | Clade 2 |
| 118 | HAdV-B3/USA/8E3/2011 | the United States | USA | 2011.5 | HAdV-B3 | OR753121 | OR753121 | OR753121 | Clade 2 | Clade 2 | Clade 2 |
| 119 | HAdV-B3/USA/2E1/2009 | the United States | USA | 2009.5 | HAdV-B3 | OR753120 | OR753120 | OR753120 | Clade 2 | Clade 2 | Clade 2 |
| 120 | HAdV-B3/USA/2E2/2009 | the United States | USA | 2009.5 | HAdV-B3 | OR753109 | OR753109 | OR753109 | Other | Clade 2 | Clade 2 |
| 121 | HAdV-B3/USA/6G8/2010 | the United States | USA | 2010.5 | HAdV-B3 | OR735201 | OR735201 | OR735201 | Other | Other | Other |
| 122 | human/USA/UFL_Adv3a51/2007/3[P3H3F3] | the United States | USA | 2007.1 | HAdV-B3 | KF268123 | KF268123 | KF268123 | Clade 2 | Clade 2 | Clade 2 |
| 123 | human/USA/UFL_Adv3a50/2007/3[P3H3F3] | the United States | USA | 2007.2 | HAdV-B3 | KF268133 | KF268133 | KF268133 | Clade 2 | Clade 2 | Clade 2 |
| 124 | human/USA/UFL_Adv3a17/2007/3[P3H3F3] | the United States | USA | 2007.2 | HAdV-B3 | KF268131 | KF268131 | KF268131 | Clade 1 | Clade 1 | Clade 1 |
| 125 | human/USA/UFL_Adv3a2/2007/3[P3H3F3] | the United States | USA | 2007.2 | HAdV-B3 | KF268120 | KF268120 | KF268120 | Clade 2 | Clade 2 | Clade 2 |
| 126 | NHRC 1276 | the United States | USA | 1997.11 | HAdV-B3 | AY599836 | AY599836 | AY599836 | Clade 1 | Clade 1 | Clade 1 |
| 127 | human/USA/ak33_AdV3a/2003/3[P3H3F3] | the United States | USA | 2003.1 | HAdV-B3 | JX423381 | JX423381 | JX423381 | Clade 2 | Clade 2 | Clade 2 |
| 128 | human/USA/ak34_AdV3a2/2008/3[P3H3F3] | the United States | USA | 2008.1 | HAdV-B3 | JX423382 | JX423382 | JX423382 | Clade 2 | Clade 2 | Clade 2 |
| 129 | human/USA/CL_46/1988/3[P3H3F3] | the United States | USA | 1988 | HAdV-B3 | KF268128 | KF268128 | KF268128 | Clade 1 | Clade 1 | Clade 1 |
| 130 | human/USA/ak32_AdV3a/2004/3[P3H3F3] | the United States | USA | 2004.7 | HAdV-B3 | JX423380 | JX423380 | JX423380 | Clade 2 | Clade 2 | Clade 2 |
| 131 | Shenzhen-2023-5-ILI-P2288 | China Shenzhen | CHN/Shenzhen | 2023.5 | HAdV-B3 | √ | √ | √ | Clade 2 | Clade 2 | Clade 2 |
| 132 | Shenzhen-2023-12-SARI-2781 | China Shenzhen | CHN/Shenzhen | 2023.12 | HAdV-B3 | × | √ | √ | - | Clade 2 | Clade 2 |
| 133 | Shenzhen-2023-12-SARI-2871 | China Shenzhen | CHN/Shenzhen | 2023.12 | HAdV-B3 | × | √ | × | - | Clade 2 | - |
| 134 | Shenzhen-2023-12-SARI-2872 | China Shenzhen | CHN/Shenzhen | 2023.12 | HAdV-B3 | × | √ | √ | - | Clade 2 | Clade 2 |
| 135 | Shenzhen-2023-12-SARI-2875 | China Shenzhen | CHN/Shenzhen | 2023.12 | HAdV-B3 | × | √ | √ | - | Clade 2 | Clade 2 |
| 136 | Shenzhen-2023-12-SARI-2876 | China Shenzhen | CHN/Shenzhen | 2023.12 | HAdV-B3 | × | √ | √ | - | Clade 2 | Clade 2 |
| 137 | Shenzhen-2023-12-SARI-2878 | China Shenzhen | CHN/Shenzhen | 2023.12 | HAdV-B3 | × | √ | √ | - | Clade 2 | Clade 2 |
| 138 | Shenzhen-2024-1-SARI-S4 | China Shenzhen | CHN/Shenzhen | 2024.1 | HAdV-B3 | × | √ | × | - | Clade 2 | - |
| 139 | Shenzhen-2024-1-SARI-S16 | China Shenzhen | CHN/Shenzhen | 2024.1 | HAdV-B3 | × | √ | × | - | Clade 2 | - |
| 140 | Shenzhen-2024-1-ILI-292 | China Shenzhen | CHN/Shenzhen | 2024.1 | HAdV-B3 | × | √ | × | - | Clade 2 | - |
| 141 | Shenzhen-2024-1-ILI-295 | China Shenzhen | CHN/Shenzhen | 2024.1 | HAdV-B3 | × | √ | √ | - | Clade 2 | Clade 2 |
| 142 | Shenzhen-2024-1-ILI-298 | China Shenzhen | CHN/Shenzhen | 2024.1 | HAdV-B3 | × | √ | √ | - | Clade 2 | Clade 2 |
| 143 | Shenzhen-2024-1-ILI-300 | China Shenzhen | CHN/Shenzhen | 2024.1 | HAdV-B3 | × | √ | √ | - | Clade 2 | Clade 2 |
| 144 | Shenzhen-2024-2-ILI-337 | China Shenzhen | CHN/Shenzhen | 2024.2 | HAdV-B3 | × | √ | × | - | Clade 2 | - |
| 145 | Shenzhen-2024-2-ILI-338 | China Shenzhen | CHN/Shenzhen | 2024.2 | HAdV-B3 | × | × | √ | - | - | Clade 2 |
| 146 | Shenzhen-2024-2-ILI-339 | China Shenzhen | CHN/Shenzhen | 2024.2 | HAdV-B3 | × | √ | √ | - | Clade 2 | Clade 2 |
| 147 | Shenzhen-2024-2-SARI-415 | China Shenzhen | CHN/Shenzhen | 2024.2 | HAdV-B3 | × | √ | √ | - | Clade 2 | Clade 2 |
| 148 | Shenzhen-2024-2-SARI-416 | China Shenzhen | CHN/Shenzhen | 2024.2 | HAdV-B3 | × | √ | √ | - | Clade 2 | Clade 2 |
| 149 | Shenzhen-2024-2-ILI-420 | China Shenzhen | CHN/Shenzhen | 2024.2 | HAdV-B3 | × | √ | √ | - | Clade 2 | Clade 2 |
| 150 | Shenzhen-2024-2-ILI-422 | China Shenzhen | CHN/Shenzhen | 2024.2 | HAdV-B3 | × | √ | × | - | Clade 2 | - |
| 151 | Shenzhen-2024-2-ILI-434 | China Shenzhen | CHN/Shenzhen | 2024.2 | HAdV-B3 | × | √ | √ | - | Clade 2 | Clade 2 |
| 152 | Shenzhen-2024-5-ILI-1019 | China Shenzhen | CHN/Shenzhen | 2024.5 | HAdV-B3 | × | √ | √ | - | Clade 2 | Clade 2 |
| 153 | Shenzhen-2024-1-F1-S0-437 | China Shenzhen | CHN/Shenzhen | 2024.1 | HAdV-B3 | √ | √ | √ | Clade 2 | Clade 2 | Clade 2 |
| 154 | Shenzhen-2024-1-F1-S0-932 | China Shenzhen | CHN/Shenzhen | 2024.1 | HAdV-B3 | √ | √ | √ | Clade 2 | Clade 2 | Clade 2 |
| 155 | Shenzhen-2024-1-F2-S0-430 | China Shenzhen | CHN/Shenzhen | 2024.1 | HAdV-B3 | √ | √ | √ | Clade 2 | Clade 2 | Clade 2 |
| 156 | Shenzhen-2024-1-F3-S0-943 | China Shenzhen | CHN/Shenzhen | 2024.1 | HAdV-B3 | √ | √ | √ | Clade 2 | Clade 2 | Clade 2 |
| 157 | Shenzhen-2024-1-F5-S1-261 | China Shenzhen | CHN/Shenzhen | 2024.1 | HAdV-B3 | √ | √ | √ | Clade 2 | Clade 2 | Clade 2 |
| 158 | Shenzhen-2024-1-F5-S1-132 | China Shenzhen | CHN/Shenzhen | 2024.1 | HAdV-B3 | √ | √ | √ | Clade 2 | Clade 2 | Clade 2 |
| 159 | Shenzhen-2024-1-F5-S0-473 | China Shenzhen | CHN/Shenzhen | 2024.1 | HAdV-B3 | √ | √ | √ | Clade 2 | Clade 2 | Clade 2 |
| 160 | Shenzhen-2024-1-F6-S0-214 | China Shenzhen | CHN/Shenzhen | 2024.1 | HAdV-B3 | √ | √ | √ | Clade 2 | Clade 2 | Clade 2 |

**Supplement Table 7.** Amino acid site variation of Hexon gene of global HAdV-B3 strains

| Strain name | Clade | Hexon | | | | | | | | | | | | | | | | | | | | | | | | | | | | | |
| --- | --- | --- | --- | --- | --- | --- | --- | --- | --- | --- | --- | --- | --- | --- | --- | --- | --- | --- | --- | --- | --- | --- | --- | --- | --- | --- | --- | --- | --- | --- | --- |
|  |  | 62 | 64 | 104 | 168 | 172 | 184 | 217 | 218 | 220 | 262 | 265 | 274 | 285 | 290 | 342 | 345 | 347 | 376 | 382 | 403 | 411 | 431 | 468 | 476 | 487 | 489 | 490 | 520 | 526 | 541 |
| CHN/Beijing-2011-B3-KM458623 | Clade 2 | T | F | R | G | K | M | T | E | P | G | D | T | I | C | A | I | L | A | G | I | E | P | L | S | M | I | H | K | K | S |
| CHN/Beijing-2012-B3-KM458624 | Clade 2 | . | . | . | . | . | . | . | . | . | . | . | . | . | . | . | . | . | . | . | . | . | . | . | . | . | . | . | . | . | . |
| CHN/Beijing-2013-B3-KM458630 | Clade 2 | . | . | . | . | . | . | . | . | . | . | . | . | . | . | . | . | . | . | . | . | . | . | . | . | . | . | . | . | . | . |
| CHN/Beijing-2015-B3-MW748602 (7 strains) | Clade 2 | . | . | . | . | . | . | . | . | . | . | . | . | . | . | . | . | . | . | . | . | . | . | . | . | . | . | . | . | . | . |
| CHN/Beijing-2015-B3-MW748608 | Clade 2 | . | . | . | . | . | . | . | . | . | . | . | . | . | . | . | . | . | . | . | . | . | . | . | . | . | . | . | . | . | . |
| CHN/Beijing-2016-B3-MW748600 | Clade 2 | . | . | . | . | . | . | . | . | . | . | . | . | . | . | . | . | . | . | . | . | . | . | . | . | . | . | . | . | . | . |
| CHN/Beijing-2016-B3-MW748601 | Clade 2 | . | . | . | . | . | . | . | . | . | . | . | . | . | . | . | . | . | . | . | . | . | . | . | . | . | . | . | . | . | . |
| CHN/Beijing-2017-B3-MW748610 (7 strains) | Clade 2 | . | . | . | . | . | . | . | . | . | . | . | . | . | . | . | . | . | . | . | . | . | . | . | . | . | . | . | . | . | . |
| CHN/Beijing-2017-B3-MW748611 | Clade 2 | . | . | . | . | . | . | . | . | . | . | . | . | . | . | . | . | . | . | . | . | . | . | . | . | . | . | . | . | . | . |
| CHN/Beijing-2017-B3-MW748612 | Clade 2 | . | . | . | . | . | . | . | . | . | . | . | . | . | . | . | . | . | . | . | . | . | . | . | . | . | . | . | . | . | . |
| CHN/Guangzhou-2004-B3-DQ105654 | Clade 2 | A | . | . | . | . | V | . | . | . | . | . | . | . | . | . | . | . | . | . | . | . | . | . | . | . | . | . | . | . | . |
| CHN/Guangzhou-2005-B3-DQ099432 | Clade 2 | . | . | . | . | . | . | . | . | . | . | . | . | . | . | . | . | . | . | . | . | . | . | . | . | . | . | . | . | . | . |
| CHN/Guangzhou-2011-B3-JQ764730 | Clade 2 | . | L | . | . | R | . | . | . | L | . | . | A | . | . | . | . | . | . | . | . | . | . | . | . | . | . | . | . | . | . |
| CHN/Guangzhou-2015-B3-MW748597 (3 strains) | Clade 2 | . | . | . | . | . | . | . | . | . | . | . | . | . | . | . | . | . | . | . | . | . | . | . | . | . | . | . | . | . | . |
| CHN/Hangzhou-2011-B3-KF551934 | Clade 2 | . | . | . | . | . | . | . | . | . | . | . | . | . | . | . | . | . | . | . | . | . | . | . | . | . | . | . | . | . | . |
| CHN/Inner Mongolia-2019-B3-MW767985 | Clade 2 | . | . | . | . | . | . | . | . | . | . | . | . | . | . | . | . | . | . | . | . | . | . | . | . | . | . | . | . | . | . |
| CHN/Shanghai-2009-B3-MK813914 (5 strains) | Clade 2 | . | . | . | . | . | . | . | . | . | . | . | . | . | . | . | . | . | . | . | . | . | . | . | . | . | . | . | . | . | . |
| Shenzhen-2024-5-ILI-1019 | Clade 2 | . | . | . | . | . | . | . | . | . | . | . | . | . | . | . | . | . | . | . | . | . | . | . | . | . | . | . | . | . | . |
| Shenzhen-2024-2-SARI-416 | Clade 2 | . | . | . | . | . | . | . | . | . | . | . | . | . | . | . | . | . | . | . | . | . | . | . | . | . | . | . | . | . | . |
| Shenzhen-2024-2-SARI-415 | Clade 2 | . | . | . | . | . | . | . | . | . | . | . | . | . | . | . | . | . | . | . | . | . | . | . | . | . | . | . | . | . | . |
| Shenzhen-2024-2-ILI-434 | Clade 2 | . | . | . | . | . | . | . | . | . | . | . | . | . | . | . | . | . | . | . | . | . | . | . | . | . | . | . | . | . | . |
| Shenzhen-2024-2-ILI-422 | Clade 2 | . | . | . | . | . | . | . | . | . | . | . | . | . | . | . | . | . | . | . | . | . | . | . | . | . | . | . | . | . | . |
| Shenzhen-2024-2-ILI-420 | Clade 2 | . | . | . | . | . | . | . | . | . | . | . | . | . | . | . | . | . | . | . | . | . | . | . | . | . | . | . | . | . | . |
| Shenzhen-2024-2-ILI-339 | Clade 2 | . | . | . | . | . | . | . | . | . | . | . | . | . | . | . | . | . | . | . | . | . | . | . | . | . | . | . | . | . | . |
| Shenzhen-2024-2-ILI-337 | Clade 2 | . | . | . | . | . | . | . | . | . | . | . | . | . | . | . | . | . | . | . | . | . | . | . | . | . | . | . | . | . | . |
| Shenzhen-2024-1-SARI-S16 | Clade 2 | . | . | . | . | . | . | . | . | . | . | . | . | . | . | . | . | . | . | . | . | . | . | . | . | . | . | . | . | . | . |
| Shenzhen-2024-1-SARI-S4 | Clade 2 | . | . | . | . | . | . | . | . | . | . | . | . | . | . | . | . | . | . | . | . | . | . | . | . | . | . | . | . | . | . |
| Shenzhen-2024-1-ILI-300 | Clade 2 | . | . | . | . | . | . | . | . | . | . | . | . | . | . | . | . | . | . | . | . | . | . | . | . | . | . | . | . | . | . |
| Shenzhen-2024-1-ILI-298 | Clade 2 | . | . | . | . | . | . | . | . | . | . | . | . | . | . | . | . | . | . | . | . | . | . | . | . | . | . | . | . | . | . |
| Shenzhen-2024-1-ILI-295 | Clade 2 | . | . | . | . | . | . | . | . | . | . | . | . | . | . | . | . | . | . | . | . | . | . | . | . | . | . | . | . | . | . |
| Shenzhen-2024-1-ILI-292 | Clade 2 | . | . | . | . | . | . | . | . | . | . | . | . | . | . | . | . | . | . | . | . | . | . | . | . | . | . | . | . | . | . |
| Shenzhen-2024-1-F6-S0-214 | Clade 2 | . | . | . | . | . | . | . | . | . | . | . | . | . | . | . | . | . | . | . | . | . | . | . | . | . | . | . | . | . | . |
| Shenzhen-2024-1-F5-S1-261 | Clade 2 | . | . | . | . | . | . | . | . | . | . | . | . | . | . | . | . | . | . | . | . | . | . | . | . | . | . | . | . | . | . |
| Shenzhen-2024-1-F5-S1-132 | Clade 2 | . | . | . | . | . | . | . | . | . | . | . | . | . | . | . | . | . | . | . | . | . | . | . | . | . | . | . | . | . | . |
| Shenzhen-2024-1-F5-S0-473 | Clade 2 | . | . | . | . | . | . | . | . | . | . | . | . | . | . | . | . | . | . | . | . | . | . | . | . | . | . | . | . | . | . |
| Shenzhen-2024-1-F3-S0-943 | Clade 2 | . | . | . | . | . | . | . | . | . | . | . | . | . | . | . | . | . | . | . | . | . | . | . | . | . | . | . | . | . | . |
| Shenzhen-2024-1-F2-S0-430 | Clade 2 | . | . | . | . | . | . | . | . | . | . | . | . | . | . | . | . | . | . | . | . | . | . | . | . | . | . | . | . | . | . |
| Shenzhen-2024-1-F1-S0-932 | Clade 2 | . | . | . | . | . | . | . | . | . | . | . | . | . | . | . | . | . | . | . | . | . | . | . | . | . | . | . | . | . | . |
| Shenzhen-2024-1-F1-S0-437 | Clade 2 | . | . | . | . | . | . | . | . | . | . | . | . | . | . | . | . | . | . | . | . | . | . | . | . | . | . | . | . | . | . |
| Shenzhen-2023-12-SARI-2878 | Clade 2 | . | . | . | . | . | . | . | . | . | . | . | . | . | . | . | . | . | . | . | . | . | . | . | . | . | . | . | . | . | . |
| Shenzhen-2023-12-SARI-2876 | Clade 2 | . | . | . | . | . | . | . | . | . | . | . | . | . | . | . | . | . | . | . | . | . | . | . | . | . | . | . | . | . | . |
| Shenzhen-2023-12-SARI-2875 | Clade 2 | . | . | . | . | . | . | . | . | . | . | . | . | . | . | . | . | . | . | . | . | . | . | . | . | . | . | . | . | . | . |
| Shenzhen-2023-12-SARI-2872 | Clade 2 | . | . | . | . | . | . | . | . | . | . | . | . | . | . | . | . | . | . | . | . | . | . | . | . | . | . | . | . | . | . |
| Shenzhen-2023-12-SARI-2871 | Clade 2 | . | . | . | . | . | . | . | . | . | . | . | . | . | . | . | . | . | . | . | . | . | . | . | . | . | . | . | . | . | . |
| Shenzhen-2023-12-SARI-2781 | Clade 2 | . | . | . | . | . | . | . | . | . | . | . | . | . | . | . | . | . | . | . | . | . | . | . | . | . | . | . | . | . | . |
| Shenzhen-2023-5-ILI-P2288 | Clade 2 | . | . | . | . | . | . | . | . | . | . | . | . | . | . | . | . | . | . | . | . | . | . | . | . | . | . | . | . | . | . |
| CHN/Shanghai-2011-B3-MK847517 (3 strains) | Clade 2 | . | . | . | . | . | . | . | . | . | . | . | . | . | . | . | . | . | . | . | . | . | . | . | . | . | . | . | . | . | . |
| CHN/Shanghai-2012-B3-MK883608 | Clade 2 | . | . | . | . | . | . | . | . | . | . | . | . | . | . | . | . | . | . | . | . | . | . | . | . | . | . | . | . | . | . |
| CHN/Shanxi-2018-B3-OQ128153 (9 strains) | Clade 2 | . | . | . | . | . | . | . | . | . | . | . | . | . | . | . | . | . | . | . | . | . | . | . | . | . | . | . | . | . | . |
| CHN/Shanxi-2019-B3-OQ128162 | Clade 2 | . | . | . | . | . | . | . | . | . | . | . | . | . | . | . | . | . | . | . | . | . | . | . | . | . | . | . | . | . | . |
| CHN/Taiwan-1964-B3-EF494642 | Clade 2 | . | . | . | . | . | . | . | . | . | . | . | . | . | . | . | . | . | . | . | . | . | . | . | . | . | . | . | . | . | . |
| CHN/Taiwan-2011-B3-KC456084 (21 strains) | Clade 2 | . | . | . | . | . | . | . | . | . | . | . | . | . | . | . | . | . | . | . | . | . | . | . | . | . | . | . | . | . | . |
| JPN-2003-B3-AB900151 | Clade 2 | . | . | . | . | . | . | . | . | . | . | . | . | . | . | . | . | . | . | . | . | . | . | . | . | . | . | . | . | . | . |
| JPN-2004-B3-AB900154 | Clade 2 | . | . | . | . | . | . | . | . | . | . | . | . | . | . | . | . | . | . | . | . | . | . | . | . | . | . | . | . | . | . |
| KOR-2015-B3-KY320276 | Clade 2 | . | . | . | . | . | . | . | . | . | . | . | . | . | . | . | . | . | . | . | . | . | . | . | . | . | . | . | . | . | . |
| USA-2002-B3-KX384958 | Clade 2 | . | . | . | . | . | . | . | . | . | . | . | . | . | . | . | . | . | . | . | . | . | . | . | . | . | . | . | . | . | . |
| USA-2003-B3-JX423381 | Clade 2 | . | . | . | . | . | . | . | . | . | . | . | . | . | . | . | . | . | . | . | . | . | . | . | . | . | . | . | . | . | . |
| USA-2004-B3-JX423380 | Clade 2 | . | . | . | . | . | . | . | . | . | . | . | . | . | . | . | . | . | . | . | . | . | . | . | . | . | . | . | . | . | . |
| USA-2004-B3-KF268195 | Clade 2 | . | . | . | . | . | . | . | . | . | . | . | . | . | . | . | . | . | . | . | . | . | . | . | . | . | . | . | . | . | . |
| USA-2007-B3-KF268120 (3 strains) | Clade 2 | . | . | . | . | . | . | . | . | . | . | . | . | . | . | . | . | . | . | . | . | . | . | . | . | . | . | . | . | . | . |
| USA-2008-B3-JX423382 | Clade 2 | . | . | . | . | . | . | . | . | . | . | . | . | . | . | . | . | . | . | . | . | . | . | . | . | . | . | . | . | . | . |
| USA-2009-B3-OQ518267 (7 strains) | Clade 2 | . | . | . | . | . | . | . | . | . | . | . | . | . | . | . | . | . | . | . | . | . | . | . | . | . | . | . | . | . | . |
| USA-2010-B3-OR876397 (6 strains) | Clade 2 | . | . | . | . | . | . | . | . | . | . | . | . | . | . | . | . | . | . | . | . | . | . | . | . | . | . | . | . | . | . |
| USA-2011-B3-OQ518278 (4 strains) | Clade 2 | . | . | . | . | . | . | . | . | . | . | . | . | . | . | . | . | . | . | . | . | . | . | . | . | . | . | . | . | . | . |
| USA-2012-B3-OQ518266 (2 strains) | Clade 2 | . | . | . | . | . | . | . | . | . | . | . | . | . | . | . | . | . | . | . | . | . | . | . | . | . | . | . | . | . | . |
| USA-2012-B3-PP068614 | Clade 2 | . | . | . | . | . | . | . | . | . | . | . | . | . | . | . | . | . | V | . | . | . | . | . | . | . | . | . | . | . | . |
| USA-2013-B3-OQ518287 (5 strains) | Clade 2 | . | . | . | . | . | . | . | . | . | . | . | . | . | . | . | . | . | . | . | . | . | . | . | . | . | . | . | . | . | . |
| USA-2015-B3-OR777196 (2 strains) | Clade 2 | . | . | . | . | . | . | . | . | . | . | . | . | . | . | . | . | . | . | . | . | . | . | . | . | . | . | . | . | . | . |
| USA-2016-B3-OR777202 | Clade 2 | . | . | . | . | . | . | . | . | . | . | . | . | . | . | . | . | . | . | . | . | . | . | . | . | . | . | . | . | . | . |
| USA-2019-B3-OR777175 (2 strains) | Clade 2 | . | . | . | . | . | . | . | . | . | . | . | . | . | . | . | . | . | . | . | . | . | . | . | . | . | . | . | . | . | . |
| USA-2020-B3-OR777156 | Clade 2 | . | . | . | . | . | . | . | . | . | . | . | . | . | . | . | . | . | . | . | . | . | . | . | . | . | . | . | . | . | . |
| CHN/Taiwan-2004-B3-EF494643 | Clade 1 | . | . | . | V | . | . | I | . | . | . | . | . | . | . | . | . | . | . | . | L | . | . | . | . | . | . | . | . | . | L |
| IND-2011-B3-KF268210 (3 strains) | Clade 1 | . | . | . | . | . | . | . | . | . | . | . | . | . | . | . | . | . | . | . | L | . | . | . | . | . | . | . | . | . | . |
| JPN-1988-B3-AB900148 | Clade 1 | . | . | . | V | . | . | . | . | . | . | . | . | . | . | . | . | . | . | . | L | . | . | . | . | . | . | . | . | . | L |
| JPN-2019-B3-LC695001 | Clade 1 | . | . | . | V | . | . | I | Q | . | . | . | . | . | . | . | . | . | . | . | L | . | . | . | . | . | . | . | . | . | L |
| USA-1988-B3-KF268128 (2 strains) | Clade 1 | . | . | . | V | . | . | . | . | . | . | . | . | . | . | . | . | . | . | . | L | . | . | . | . | . | . | . | . | . | L |
| USA-1997-B3-AY599836 | Clade 1 | . | . | . | V | . | . | . | . | . | . | . | . | . | . | . | . | . | . | . | L | . | . | . | . | . | . | . | . | . | L |
| USA-2003-B3-KF268202 | Clade 1 | . | . | . | V | . | . | . | . | . | . | . | . | . | . | . | . | . | . | . | L | . | . | . | . | . | . | . | . | . | L |
| USA-2007-B3-KF268131 | Clade 1 | . | . | . | V | . | . | . | . | . | . | . | . | . | . | . | . | . | . | . | L | . | . | . | . | . | . | . | . | . | L |


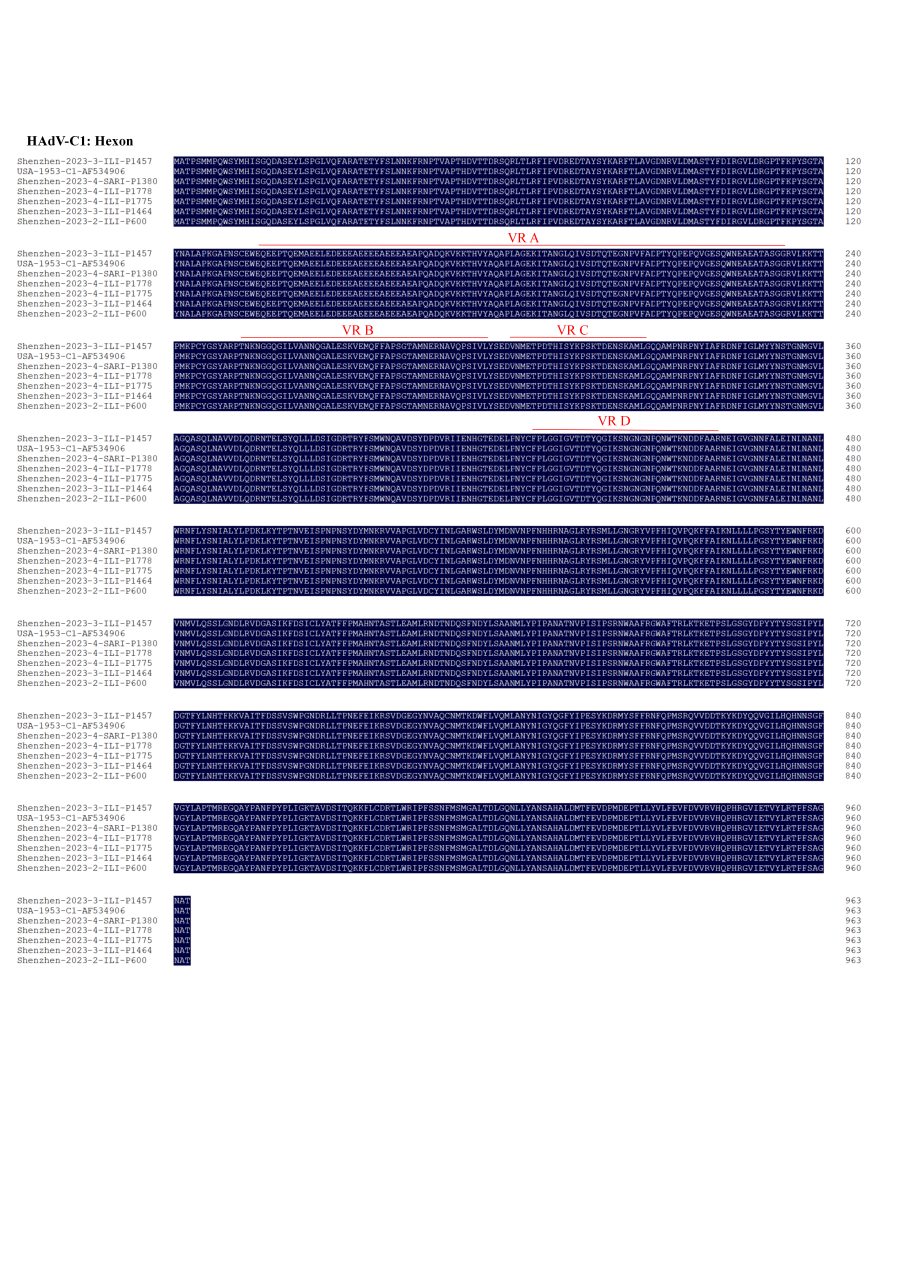


**Supplement Fig. 1.** Multiple sequence alignment of HAdV-C1 Hexon proteins. Four hypervariable regions of Hexon were shown. The number showed the position of amino acid in the protein.


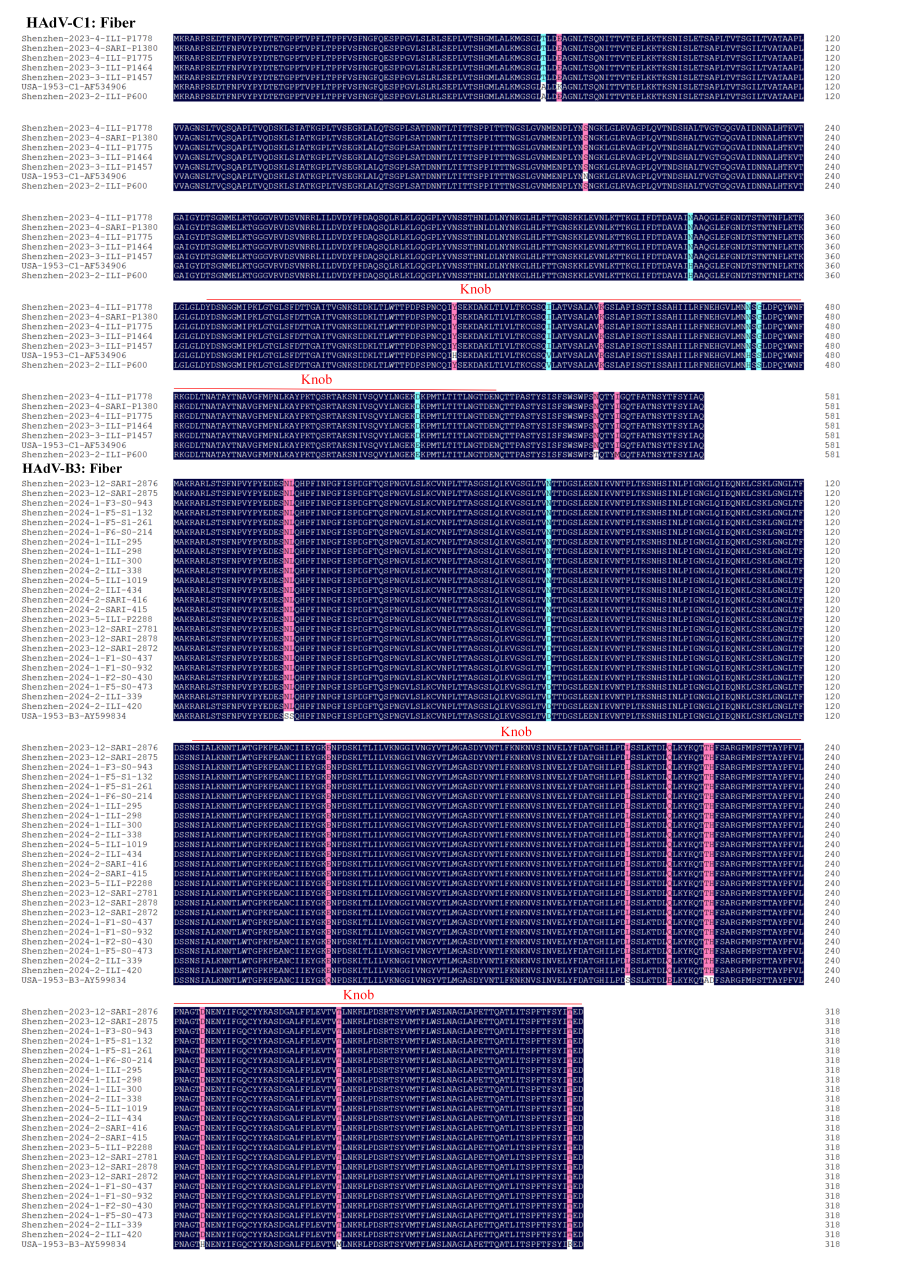


**Supplement Fig. 2.** Multiple sequence alignment of HAdV-C1 and HAdV-B3 Fiber proteins. Knob region of Fiber were shown. The number showed the position of amino acid in the protein.


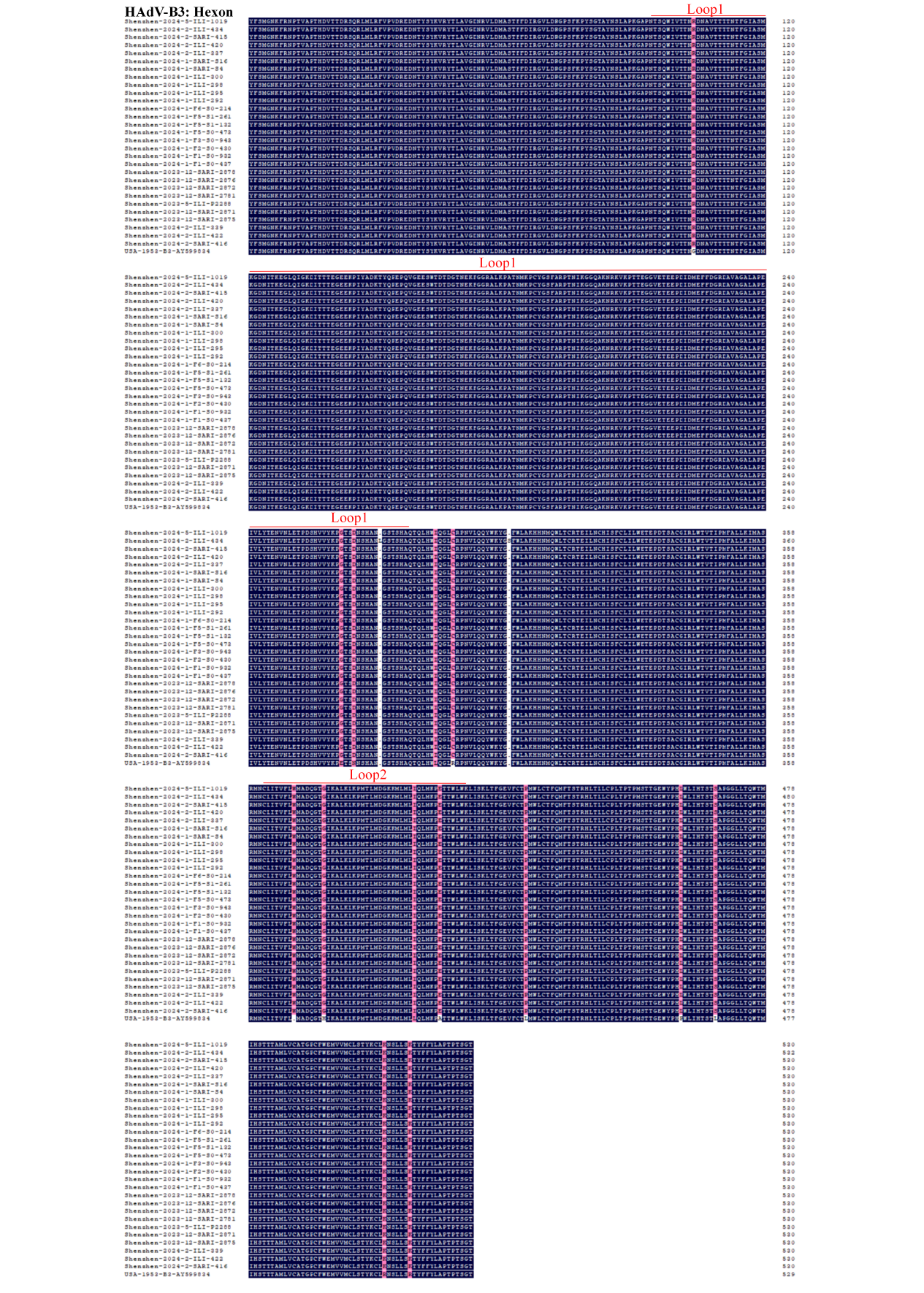


**Supplement Fig. 3.** Multiple sequence alignment of HAdV-B3 Hexon proteins. Loop1 and loop2 regions of Hexon were shown. The number showed the position of amino acid in the protein.
